# MKL-1-induced PINK1-AS overexpression contributes to the malignant progression of hepatocellular carcinoma via ALDOA-mediated glycolysis

**DOI:** 10.1038/s41598-022-24023-w

**Published:** 2022-12-09

**Authors:** Jun Wang, Hui-Min Zhang, Zhou-Tong Dai, You Huang, Hui Liu, Zhen Chen, Yuan Wu, Xing-Hua Liao

**Affiliations:** 1 Institute of Biology and Medicine, College of Life and Health Sciences, Wuhan University of Science and Technology, Wuhan, 430081 Hubei People’s Republic of China; 2 Hubei Cancer Hospital, Tongji Medical College, Huazhong University of Science and Technology, Wuhan, 430079 Hubei China; 3 Department of Radiation Oncology, Hubei Cancer Hospital, Tongji Medical College, Huazhong University of Science and Technology, Wuhan, 430079 Hubei China

**Keywords:** Cancer, Cell biology

## Abstract

Aldolase A (ALDOA), an important metabolic enzyme in the glycolytic pathway, plays an important role in regulating tumour metabolism. In this study, we investigated the expression pattern of ALDOA in hepatocellular carcinoma (HCC) and its biological role in tumour progression. Bioinformatics analysis, western blot (WB) and RT-qPCR were performed to detect the relative expression of ALDOA in HCC tissues and cell lines. A loss-of-function approach was used to investigate the biological function of ALDOA. The role of ALDOA on glycolysis was assessed by WB, glucose and lactate assay kits and a nude mouse xenograft model. Luciferase reporter experiment, chromatin immunoprecipitation and WB were performed to elucidate the underlying molecular. The expression level of ALODA was up-regulated in HCC tissues and cell lines. High ALDOA levels were associated with poorer patient overall survival. Mechanistic studies suggest that ALDOA is a direct target of miR-34a-5p, which can inhibit glycolysis in hepatocellular carcinoma cells by targeting the 3′UTR of ALDOA. PINK1 antisense RNA (PINK1-AS) competitively sponged miR-34a-5p to increase ALDOA expression by antagonizing miR-34a-5p-mediated ALDOA inhibition. MKL-1 acted as a transcription factor to promote the expression of PINK1-AS and ALDOA, thus promoting the deterioration of HCC cells. This study shows that high expression of ALDOA contributes to the development and poor prognosis of hepatocellular carcinoma and will be a target and potential prognostic biomarker for the treatment of HCC.

## Introduction

Hepatocellular carcinoma (HCC) is one of the most common malignant tumours worldwide, with the sixth and fourth highest incidence and mortality rates in the world respectively^1^. With the development of modern science and medical technology, new treatment methods have emerged, but the overall treatment effect of HCC has not been significantly improved^2^. Due to the uncertainty of the tumour location, untimely diagnosis and the ease of metastasis, patients may easily miss the best time for treatment, and the prognosis is often poor^3,4^. Therefore, the search for biomarkers and therapeutic targets of HCC has important clinical value.

Aerobic glycolysis (Warburg effect) is one of the most important metabolic changes in tumours^5^. In contrast to normal cells, tumours have altered energy production patterns and key enzymes of glycolysis can also act as signalling molecules to regulate important tumour-related signalling pathways, altering the proliferation and invasive capacity of tumour cells^6,7^. Aldolase A (ALDOA) is an important metabolic enzyme in the glycolytic and gluconeogenic pathways, thereby regulating the body's energy metabolism. Current studies have shown that ALDOA is abnormally expressed in many cancers, such as Lung adenocarcinoma and colorectal cancer^8,9^, and that ALDOA expression is significantly increased in the serum of some tumour patients, suggesting that ALDOA may be a key molecule in tumour development and malignancy. These studies suggest that ALDOA may be a potential marker for the diagnosis of a variety of tumours and a new target for targeted drug therapy, providing an effective approach for the treatment of HCC.

MicroRNAs (miRNAs) are a class of non-coding RNAs with regulatory functions and are 20–25 nucleotides in length. miRNAs can cluster with miRNA response elements (MREs) in the 3′UTR region of mRNAs to facilitate degradation or translation of mRNAs into proteins^10^. In recent years, it has been found that miRNA expression is associated with a variety of malignant tumours, and it can directly or indirectly activate or inhibit the expression of proto-oncogenes/oncogenes, thus affecting the proliferation, apoptosis, invasion and metastasis of tumour cells, playing a crucial role in the process of tumour development^11,12^. MiR-34a-5p has been reported to be dysregulated in gastric, colorectal and breast cancers. Our study suggests that miR-34a-5p has an important role in regulating HCC. However, the intrinsic biological function and induction mechanism of miR-34a-5p in HCC remains unknown.

Long-stranded non-coding RNA (lncRNA) refers to RNA with transcripts longer than 200 bases and does not encode proteins^13^. LncRNA has been shown in recent years to regulate gene expression at multiple epigenetic, transcriptional and post-transcriptional levels, affecting a variety of biological and pathological processes in cells^14–16^. PINK1-AS has been shown to be closely associated with the development of various diseases such as glucose metabolism disease, Parkinson's disease and breast cancer^17–19^. Our research shows that PINK1-AS regulated ALDOA signaling by functioning as a competing endogenous RNA (ceRNA), which suppressed the degradation of ALDOA mRNA by competing with miR-34a-5p.

Transcription factors are trans-acting factors involved in the regulation of transcription in eukaryotic cells, capable of binding directly or indirectly to specific cis-acting elements to regulate the transcription of genes^20^. MKL-1 belongs to the MRTF family of transcription factors, which are multifaceted transcriptional regulators involved in the pathogenesis of multiple human diseases^21^. In the current study, we found that ALDOA was upregulated in HCC tissues and acted as an independent predictor of overall survival. Furthermore, we investigated its effect on glycolysis by regulating ALDOA expression in HCC through the PINK1-AS/miR-34a-5p axis. PINK1-AS and ALDOA are direct transcriptional targets of MKL-1, which can directly or indirectly induce the overexpression of ALDOA to inhibit glycolysis in HCC cells. These findings may provide promising biomarkers and valuable therapeutic strategies for the treatment of HCC patients.

## Materials and methods

### Cell lines

Human HCC lines Huh-7, HepG2, Huh-1, and Hep3B were obtained from the Cell Bank of the Chinese Academy of Sciences (Shanghai, China). Human liver epithelial THLE-3 cells were obtained from the American Type Culture Collection (ATCC, USA). They were cultured in DMEM or 1640 medium supplemented with 10% fetal bovine serum (FBS) at 37 °C in an incubator with 5% CO_2_. All cell lines were STR-identified and Mycoplasma testing.

### Patients and tissue samples

We collected fresh tumor tissue and paracancerous tissue from 20 HCC patients surgically resected in Hubei Cancer Hospital from January 2018 to December 2020. All tumors were classified according to the 2010 American Joint Committee on Cancer (AJCC) staging system. All patients did not receive chemotherapy or radiotherapy before surgery, and signed a written informed consent. The study was conducted in accordance with the Declaration of Helsinki and approved by the ethics review committee of Hubei Cancer Hospital.

### Western blotting (WB)

Cells were lysed on ice with RIPA lysis buffer (Beyotime, Shanghai, China) containing PMSF, and protein concentrations were determined using the Enhanced BCA Protein Assay Kit (Meilune, Wuhan, China). Protein extracts (40 μg) were separated by electrophoresis on 12.5% SDS–polyacrylamide gels, then transferred to nitrocellulose membranes and incubated overnight with antibodies together. Secondary antibodies were added and incubated for 1 h. TBST washes were used for development. The reaction was completed using the Intelligent Chemi-TM Analysis System (Thermo Fisher Scientific, Massachusetts, USA). The following antibodies were used: Anti-β-actin (1:1000, AC026, ABclonal, Wuhan, China), Anti-ALDOA (1:1000, A1142, ABclonal, Wuhan, China), Anti-MKL-1 (1:1000, A8504, ABclonal, Wuhan, China), Anti-GLUT1 (1:1000, A11208, ABclonal, Wuhan, China), Anti-PFKM (1:1000, A5477, ABclonal, Wuhan, China). Anti-HIF-1α (1:1000, A22041, ABclonal, Wuhan, China).

### RT-qPCR analysis

Expression levels of ALDOA and other genes in HCC tissues and cells were measured using qRT-PCR according to the manufacturer's instructions (Yeasen, Shanghai, China). β-actin was used as a control. Primers are listed in Supplementary Table 1. Total RNA was extracted using an ultra-pure RNA kit (CWBIO, Beijing, China) and cDNA was synthesized using a HiScript II Q RT SuperMix for qPCR kit (Vazyme, Nanjing, China).

For miRNA quantification, Bulge-loop™ miRNA qRT-PCR primer sets (one RT primer and one pair of qRT-PCR primers per set) were designed by RiboBio (Guangzhou, China) specifically for miR-34a-5p. The cDNA was synthesized using the miRNA 1st Strand cDNA Synthesis Kit (Vazyme, Nanjing, China).

### Transfection

Negative control siRNA (si-NC), siRNA-ALDOA (si-ALDOA), siRNA-PINK1-AS (si-PINK1-AS), siRNA-MKL-1 (si-MKL-1), Negative control mimic (NC mimic), miR- 34a-5p mimic (mimic), Negative control inhibitor (NC inhibitor), miR-34a-5p inhibitor (inhibitor) were purchased from Ribo Biotechnology Co., Ltd. Primers are listed in Supplementary Table 2. All plasmids were extracted with EndoFree Plasmid Midi Kit (CWBIO, Beijing, China), and all siRNAs, miRNA mimic, miRNA inhibitor and plasmids were transfected with lipofectamine 2000 (Invitrogen, Shanghai, China) according to the manufacturer's instructions.

### Glucose and lactate determination

The glucose test kit and the lactic acid test kit (Biovision, San Francisco, USA) were used to measure the concentration of glucose and lactic acid in the cell culture medium according to the manufacturer's protocol. Take the cell culture supernatant after 48 h of transfection into a 96-well plate, and make 5 replicate wells in each group. Add 10 μL of a certain concentration of working solution to each well and incubate at 37 °C for 30 min. Detect its *OD* value at the specific wavelength required by the kit.

### Lentiviral transfection and stable cell line construction

We purchased a knockdown ALDOA lentivirus from Tsingke (Beijing, China). The lentivirus was transfected into HCC cells with 5 mg/ml polyethylene for 48 h. Use puromycin (2 μg/ml) to select stable cell clones for 1 week. The knockdown efficiency was tested by WB and RT-qPCR. Sequences are listed in Supplementary Table 2.

### Luciferase reporter experiment

The cells harvested 24 h after transfection were lysed with 1× Passive Lysis Buffer (Promega) for 30 min on ice. After centrifugation, 10 μL of the supernatant was taken, and 100 μL of Luciferase Assay Buffer was added to it to measure the fluorescence value. Take the same volume of supernatant to measure the protein concentration, and finally calculate the fluorescence value per unit protein concentration.

### Chromatin immunoprecipitation

The chromatin immunoprecipitation (ChIP) experiment was performed using the EpiQuik ChIP kit (Epigentek) according to the manufacturer's instructions. Huh-7 and HepG2 cells were treated with formaldehyde and incubated for 10 min to produce DNA–protein crosslinks. Cell lysates are then sonicated to produce 200–300 bp chromatin fragments and immunoprecipitated using MKL-1 specific antibody or IgG as a control. The precipitated chromatin DNA is recovered and analyzed by RT-qPCR. The primers related to ChIP analysis are listed in Supplementary Table 3.

### Hematoxylin–eosin staining and immunohistochemistry experiment

The tumor tissues of nude mouse were sent to Servicebio Biotech for paraffin-embedded sectioning. Hematoxylin–Eosin staining kit (Solarbio, Beijing, China) is used for hematoxylin–eosin staining (H&E). Use streptavidin alkaline phosphatase immunohistochemical staining kit (Servicebio, Wuhan, China) for immunohistochemistry (IHC) experiments. All the above operations are carried out in strict accordance with the manufacturer's instructions.

### Tumor formation assay in nude mouse model

The animal experiment procedure has been approved by the Laboratory Animal Center of Wuhan University of Science and Technology and the Experimental Animal Ethics Review Committee. BALB/c-nu (nude mouse) mice obtained from Wuhan Shulb. Three-week-old nude mouse were randomly divided into two groups, each of which was injected subcutaneously with 1 × 10^8^ Huh-7 cell line that stably knocks down ALDOA and the control group. After 28 days, all nude mice were euthanized by intraperitoneal injection of sodium pentobarbital and diazepam. And check the growth of subcutaneous tumors in nude mouse.

### Ethics approval

The study was approved and reviewed by the Medical Ethics Committee of Wuhan University of Science and Technology and was conducted with the consent of the subjects and in accordance with the Declaration of Helsinki. All animal experiments were performed in compliance with the ARRIVE guidelines. The study was approved by the Animal Ethics Committee of Wuhan University of Science and Technology. All methods were carried out in accordance with relevant guidelines and regulations.

### Statistical analysis

All experiments in this article were repeated at 3 times. These values are expressed as mean ± standard deviation. Differences between various groups were evaluated using a two-tailed Student’s t-test. Kaplan–Meier survival chart was used to draw the survival curve, and the log-rank test was used for testing. A Pearson correlation analysis was performed to analyze the correlation between ALDOA, miR-34a-5p, ALDOA and MKL-1 levels. Two-sided *P* values < 0.05 were considered statistically significant.

## Results

### Expression of ALDOA is up-regulated in human HCC tissues and correlates with poor prognosis

**Figure 1:**
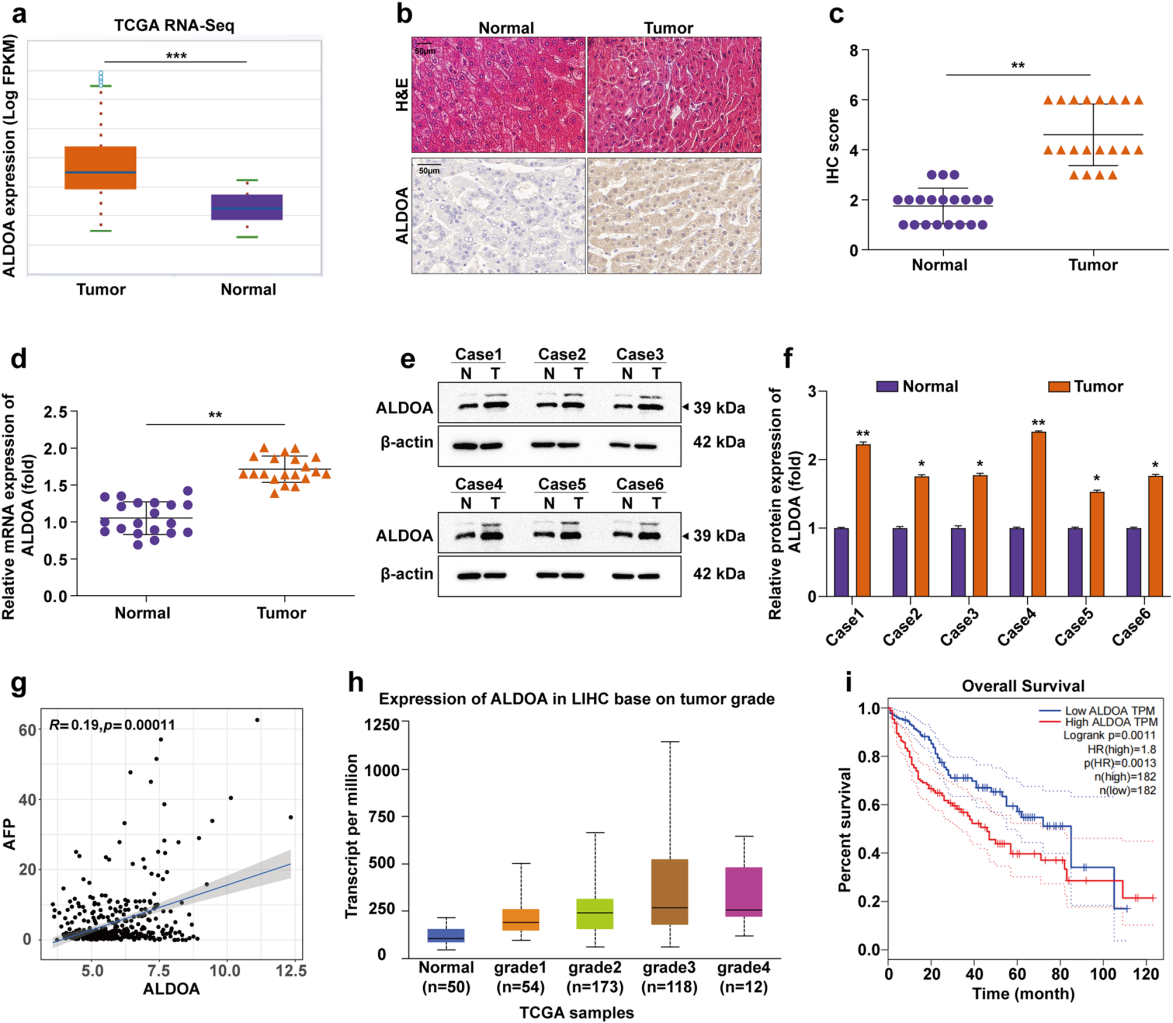
Up-regulation of ALDOA and its prognostic significance in patients with HCC. (**a**) The box diagram shows the level of ALDOA mRNA in the Starbase database. (**b**,**c**) H&E staining of the HCC tumor tissues and para-carcinoma tissues. Analysis of ALDOA distribution in tumor tissues by using IHC. Quantitative IHC analysis of ALDOA. (**d**) Relative levels of ALDOA mRNA in HCC tissues (n = 20). (**e**,**f**) Validation of ALDOA dys-regulation in HCC by using WB analysis (n = 6). Statistical data are shown. (**g**) Expression level of ALDOA was positively correlated with AFP expression level (*P* < 0.001). (**h**) Higher ALDOA levels were positively correlated with cancer stage. (**i**) Kaplan–Meier survival curves showing the effect of ALDOA on overall survival. *P < 0.05; **P < 0.01; ***P < 0.001.

We assessed ALDOA transcript levels based on the Starbase database (http://starbase.sysu.edu.cn/index.php) in multiple HCC studies. The results showed that mRNA levels of ALDOA were elevated in HCC tissues relative to normal tissues (Fig. 1a). Analysis of 15 HCC cohorts in the HCCDB database (http://lifeome.net/database/hccdb/home.html) also revealed that ALDOA mRNA expression was significantly higher in HCC tissues than in adjacent normal tissues (Fig. S1). The H&E staining was performed to confirm the morphological characteristics of HCC tissues and paracancerous tissues. IHC was used to detect the distribution of ALDOA in tumour and paraneoplastic tissues, and it was found that ALDOA was mainly expressed in HCC tissues and to a lesser extent in paraneoplastic tissues (Fig. 1b,c). Subsequently, ALDOA protein was measured by WB analysis and ALDOA mRNA was determined by RT-qPCR. The expression pattern of ALDOA was validated in 20 HCC patients, and ALDOA protein and mRNA showed an overall upward trend (Fig. 1d–f). High expression of ALDOA was found to be associated with poor prognosis in HCC patients by bioinformatics analysis. The expression level of ALDOA was positively correlated with AFP expression level (Fig. 1g). In addition, there is a significant positive correlation between higher ALDOA levels and cancer stages (Fig. 1h). As shown in Fig. 1i, elevated ALDOA levels predicted a poorer overall survival (OS) in patients with HCC.

### ALDOA is required for malignant behaviors in HCC cells

**Figure 2:**
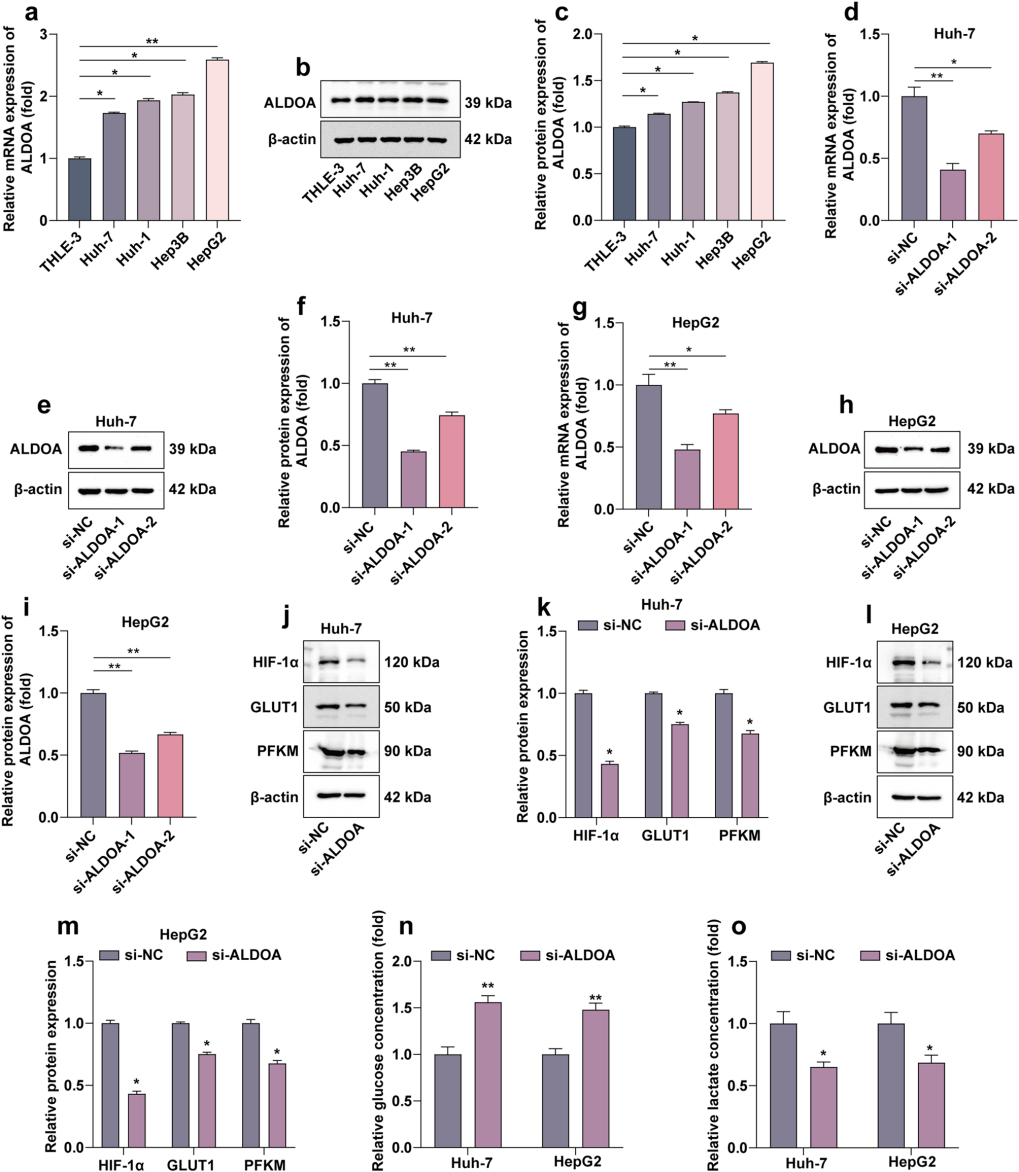
Knockdown of ALDOA inhibits aerobic glycolysis of HCC in vitro. (**a**) RT-qPCR assays for mRNA expression levels of ALDOA in normal hepatocytes and HCC cells. (**b**,**c**) WB assays for protein expression levels of ALDOA in normal hepatocytes and HCC cells. Statistical data are shown. (**d**–**i**) The effect of knockdown efficiency of ALDOA on mRNA and protein levels was validated in Huh-7 and HepG2 cells. Statistical data are shown. (**j**–**m**) WB detection of protein expression of ALDOA after knockdown and aerobic glycolysis-related marker genes in two types of HCC cells. Statistical data are shown. (**n**,**o**) Glucose and lactate levels in the culture medium supernatants of two types of HCC cells after knockdown of ALDOA were detected by the kit. *P < 0.05; **P < 0.01; ***P < 0.001.

We examined the protein and mRNA expression levels of ALDOA in the normal human hepatocyte line THLE-3 and four types of HCC cells using RT-qPCR and WB. The results showed that the expression level of ALDOA in HCC cell line was higher than that in normal cell line (Fig. 2a–c). To assess the function of ALDOA in HCC, we identified the top 50 genes positively associated with ALDOA in HCC (Fig. S2a). The functional enrichment analysis revealed that ALDOA was significantly associated with glucose metabolic processes (Fig. S2b). We next investigated the effect of ALDOA knockdown on HCC at the cellular level. SiRNA-1 sequences targeting ALDOA showed the most pronounced knockout effect and was therefore selected for subsequent experiments (Fig. 2d–i). We found that ALDOA silencing significantly inhibited the expression of aerobic glycolysis-related proteins (Fig. 2j–m). We also analysed the levels of glucose and lactate in the culture medium supernatant and found that ALDOA knockdown also significantly inhibited glucose uptake and lactate production (Fig. 2n,o). This indicated that ALDOA silencing significantly inhibited aerobic glycolysis in HCC cells.

### ALDOA promotes HCC cell tumorigenesis in vivo

**Figure 3:**
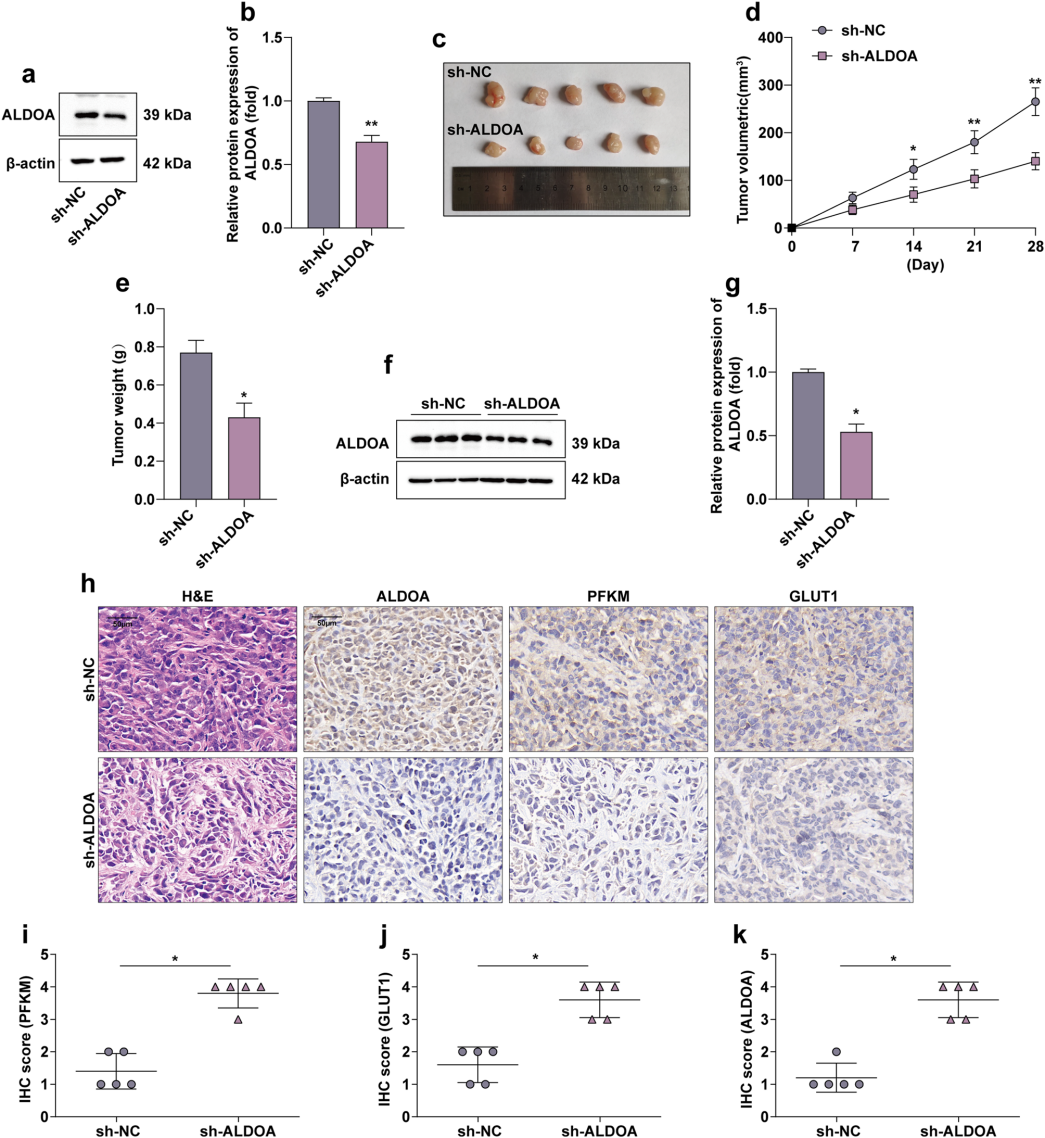
ALDOA promotes HCC cell tumorigenesis in vivo. (**a**,**b**) The knockdown efficiency of shRNA targeting ALDOA was detected by WB. Statistical data are shown. (**c**) Huh-7 cells transfected with Ctrl shRNA and ALDOA shRNA were injected respectively into nude mouse (n = 5), which were killed after 28 days. (**d**,**e**) Tumor weights and volumes were represented as the means of tumor weights ± SD. (**f**,**g**) WB results showing the protein expression levels of ALDOA in nude mouse tumour tissues. Statistical data are shown. (**h**–**k**) The tumor sections underwent IHC staining and H&E staining. Statistical data are shown. *P < 0.05; **P < 0.01.

To determine the in vivo effect of ALDOA on HCC growth, we designed shRNA based on the sequence of siRNA-1 targeting ALDOA to knockdown ALDOA (Fig. 3a,b). In a nude mouse xenograft model, Huh-7 cells with a lentiviral vector producing shRNA against ALDOA were inoculated subcutaneously into mouse, and Huh-7 cells infected with a lentiviral vector bearing a control shRNA were inoculated subcutaneously into another group of mouse as a control. Through this in vivo experiment, we observed that stable knockdown of ALDOA effectively inhibited tumour growth in nude mouse (Fig. 3c). This is reflected in a significant reduction in tumor size and weight (Fig. 3d,e). The expression of ALDOA in tumor tissues was examined by WB, which showed that ALDOA remained stably knockdown in nude mice (Fig. 3f,g). We performed histopathological analysis of tumors in nude mouse using H&E staining, and then examined the expression levels of ALDOA, PFKM and GLUT1 in nude mouse tumors by IHC. The results showed that the expression levels of ALDOA, PFKM and GLUT1 were down-regulated in sh-ALDOA group, and H&E staining showed similar change (Fig. 3h–k).

### MiR-34a-5p targets ALDOA to exert tumor suppressive effects in HCC

**Figure 4:**
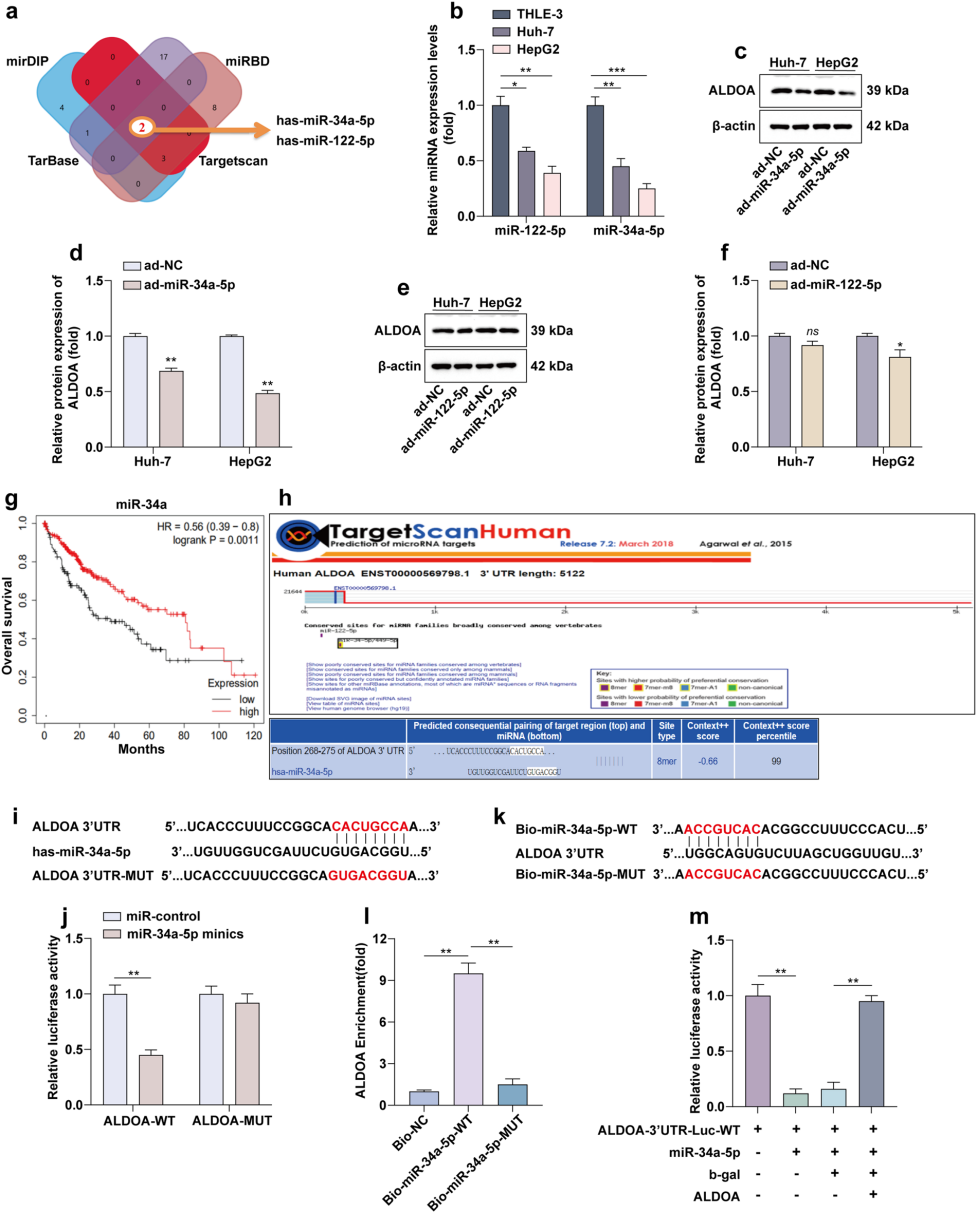
MiR-34a-5p targets ALDOA to exert tumor suppressive effects in HCC. (**a**) Venn diagram showing the 2 miRNAs predicted by four different algorithms to target ALDOA. (**b**) RT-qPCR was used to detect the expression of miR-34a-5p and miR-122-5p in Huh-7 and Hepg2 cells. (**c**–**f**) WB detection of ALDOA protein expression levels after overexpression of miR-34a-5p and miR-122-5p. Statistical data are shown. (**g**) Kaplan–Meier survival curves showing the effect of miR-34a on overall survival. (**h**,**i**) Putative binding sequence of miR-34a-5p in the 3′UTR of ALDOA. (**j**) Luciferase reporter experiment revealed that miR-34a-5p could bind to the 3′UTR of ALDOA. (**k**,**l**) Enrichment of USP4 in HCC cells transfected with miR-34a-5p-WT and miR-34a-5p-MUT plasmids. (**m**) Luciferase reporter experiment on 293 cells co-transfected with miR-34a-5p mimic or mimic-NC and ALDOA-3′UTR-Luc-WT plasmid, with or without ALDOA. *P < 0.05; **P < 0.01; ***P < 0.001.

To find miRNAs targeting ALDOA, we performed bioinformatics analysis using four different algorithms including mirDIP, Targetscan, miRDB and TarBase. Figure 4a shows the overlapping miRNAs targeting ALDOA. Expression analysis showed that miR-34a-5p was more significantly downregulated in HCC cell lines (Fig. 4b). Next we overexpressed miR-122-5p and miR-34a-5p in two HCC cell lines, respectively. The results showed that miR-34a-5p inhibited ALDOA more significantly (Fig. 4c–f). Patients with high expression of miR-34a had higher overall survival than those with low expression of miR-34a (Fig. 4g). Correlation analysis suggested a negative correlation between the level of miR-34a-5p and ALDOA expression level in HCC tissue specimens (Fig. S3a). So we chose miR-34a-5p for further study. We found the ALDOA 3′UTR binding site to miR-34a-5p, and then we mutated this binding site to construct the pmirGLO-ALDOA 3′UTR-MUT vector (Fig. 4h,i). Luciferase report experiment gene assays showed that miR-34a-5p mimics significantly reduced the luciferase activity of pmirGLO-ALDOA 3′UTR wild-type (WT), but had no effect on pmirGLO-ALDOA 3′UTR mutation (MUT) activity (Fig. 4j). Further biotin-labeled miRNA pull-down assay demonstrated that it was miR-34a-5p-WT, instead of miR-34a-5p-MUT, that enriched ALDOA (Fig. 4k,l). After transfection of ALDOA expression plasmid, the decreased luciferase activity after transfection of miR-34a-5p was restored (Fig. 4m). In addition, overexpression of miR-34a-5p inhibited the aerobic glycolysis process of HCC cells (Fig. S3b–e).

### PINK1-AS targets miR-34a-5p to exert its tumor-promoting effects in HCC

**Figure 5:**
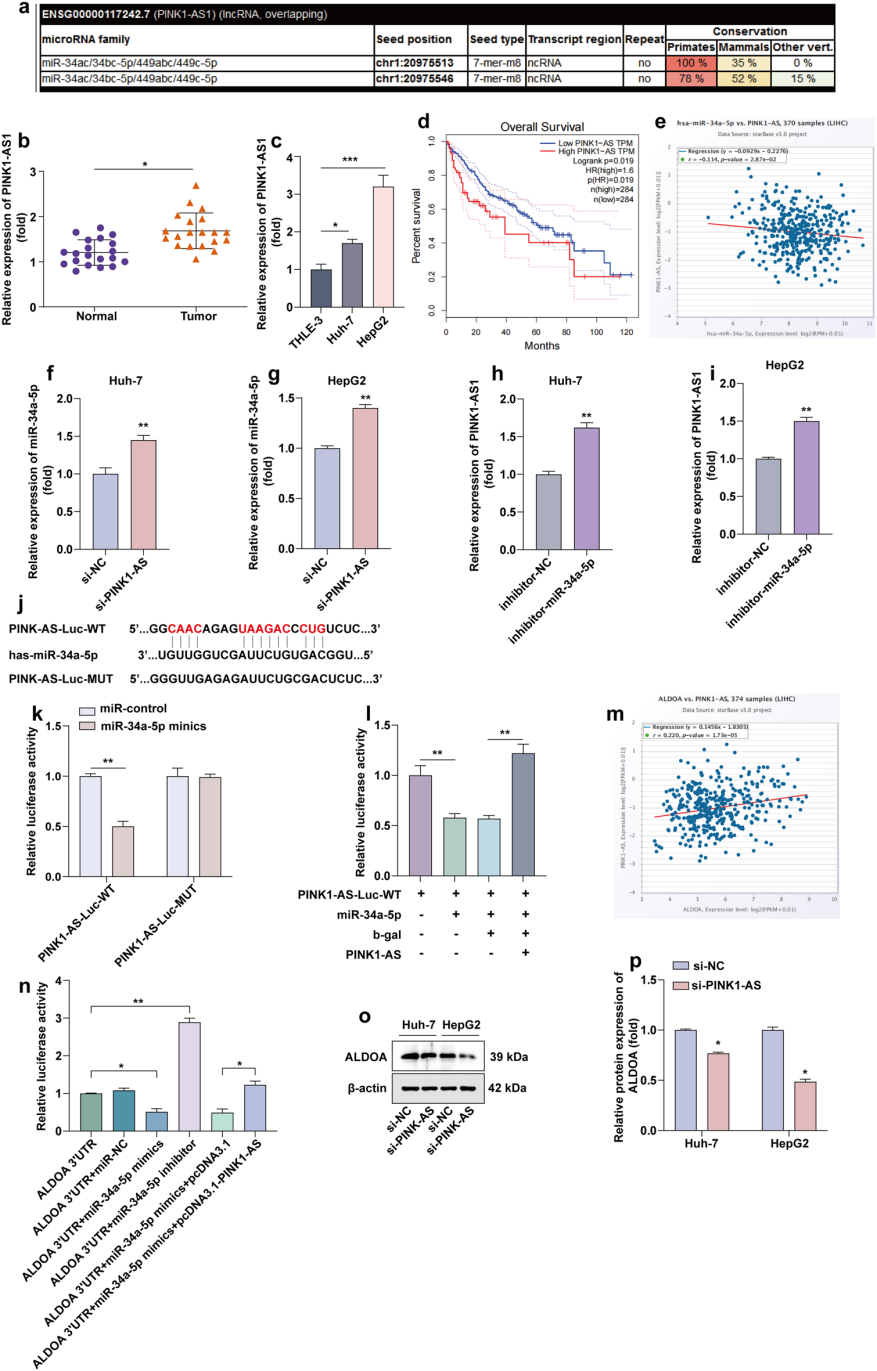
PINK1-AS targets miR-34a-5p to exert its tumor-promoting effects in HCC. (**a**) Analysis of the miRcode (http://www.mircode.org/) website shows that PINK1-AS could bind miR-34a-5p. (**b**) Relative levels of PINK1-AS mRNA in HCC tissues (n = 20). (**c**) The expression of PINK1-AS in hepatic epithelial cell and HCC cells was detected by RT-qPCR. (**d**) Kaplan–Meier survival curves showing the effect of PINK1-AS on overall survival. (**e**) Expression level of PINK1-AS was negative correlated with miR-34a-5p expression level (*P* < 0.001). (**f**,**g**) The expression level of miR-34a-5p was detected in Huh-7 cells and HepG2 cells after transfection of si-PINK1-AS by RT-qPCR. (**h**,**i**) The expression level of PINK1-AS was detected in Huh-7 cells and HepG2 cells after transfection of inhibitor-miR-34a-5p by RT-qPCR. (**j**) Putative binding sequence of miR-34a-5p in the PINK1-AS. (**k**) Luciferase reporters containing WT and MUT PINK1-AS transcript was co-transfected with miR-34a-5p mimics or miR-control in 293 cells. Luciferase activity was determined using luciferase reporter system. (**l**) Luciferase reporters containing PINK1-AS-Luc-WT were co-transfected with or without miR-34a-5p and PINK1-AS, Luciferase activity was determined using dual luciferase reporter system. (**m**) Expression level of PINK1-AS was positively correlated with ALDOA expression level (*P* < 0.001). (**n**) Relative luciferase activity of ALDOA 3′UTR was determined after transfection with miR-34a-5p mimics, miR-34a-5p inhibitor or PINK1-AS plasmid. (**o**,**p**) Down-regulation of ALDOA levels in PINK1-AS-silenced Huh-7 cells and HepG2 cells was detected by WB. Statistical data are shown. *P < 0.05; **P < 0.01; ***P < 0.001.

Analysis using miRcode (http://www.mircode.org/) website suggested that PINK1-AS could bind miR-34a-5p (Fig. 5a). We examined the expression of PINK1-AS in the 20 pairs of specimens, which was significantly up-regulated in HCC tissues (Fig. 5b). And we performed RT-qPCR to quantify the expression levels of PINK1-AS in the hepatic epithelial cell and two HCC cells. As shown in Fig. 5c, PINK1-AS was highly expressed in HCC cells, and the overall survival rate of patients with high PINK1-AS expression was poor (Fig. 5d). The expression of PINK1-AS and miR-34a-5p showed a negative correlation in tumor samples (Fig. 5e). In HCC cells, we found that knockdown the expression of PINK1-AS leads to up-regulation of miR-34a-5p (Fig. 5f,g). Inhibition of miR-34a-5p expression leads to up-regulation of PINK1-AS (Fig. 5h,i). In addition, we generated mutant sequences of PINK1-AS that were unable to bind miR-34a-5p for subsequent fluorophore enzyme reporter gene assays (Fig. 5j). As shown in Fig. 5k, miR-34a-5p mimics significantly reduced luciferase activity in HCC cells transfected with the PINK1-AS-WT sequence, whereas luciferase activity was not significantly altered in PINK1-AS-WUT transfected HCC cells. After transfection of PINK1-AS expression plasmid, the decreased luciferase activity after transfection of miR-34a-5p was restored (Fig. 5l). Bioinformatics analysis shows that the expression of PINK1-AS and ALDOA is positively correlated in tumor samples (Fig. 5m). Subsequently, the 3′UTR of ALDOA was co-transfected with the PINK1-AS plasmid and miR-34a-5p mimics. PINK1-AS overexpression negated the decrease in luciferase activity induced by overexpressing miR-34a-5p (Fig. 5n). This result implied that PINK1-AS bound to miR-34a-5p and released ALDOA from miR-34a-5p. Knockdown of PINK1-AS expression will result in down-regulation of ALDOA levels (Fig. 5o,p), and inhibited the aerobic glycolysis process of tumor cells (Fig. S4a–d).

### MKL-1 induces the expression of PINK1-AS and ALDOA by acting as a transcription factor

**Figure 6:**
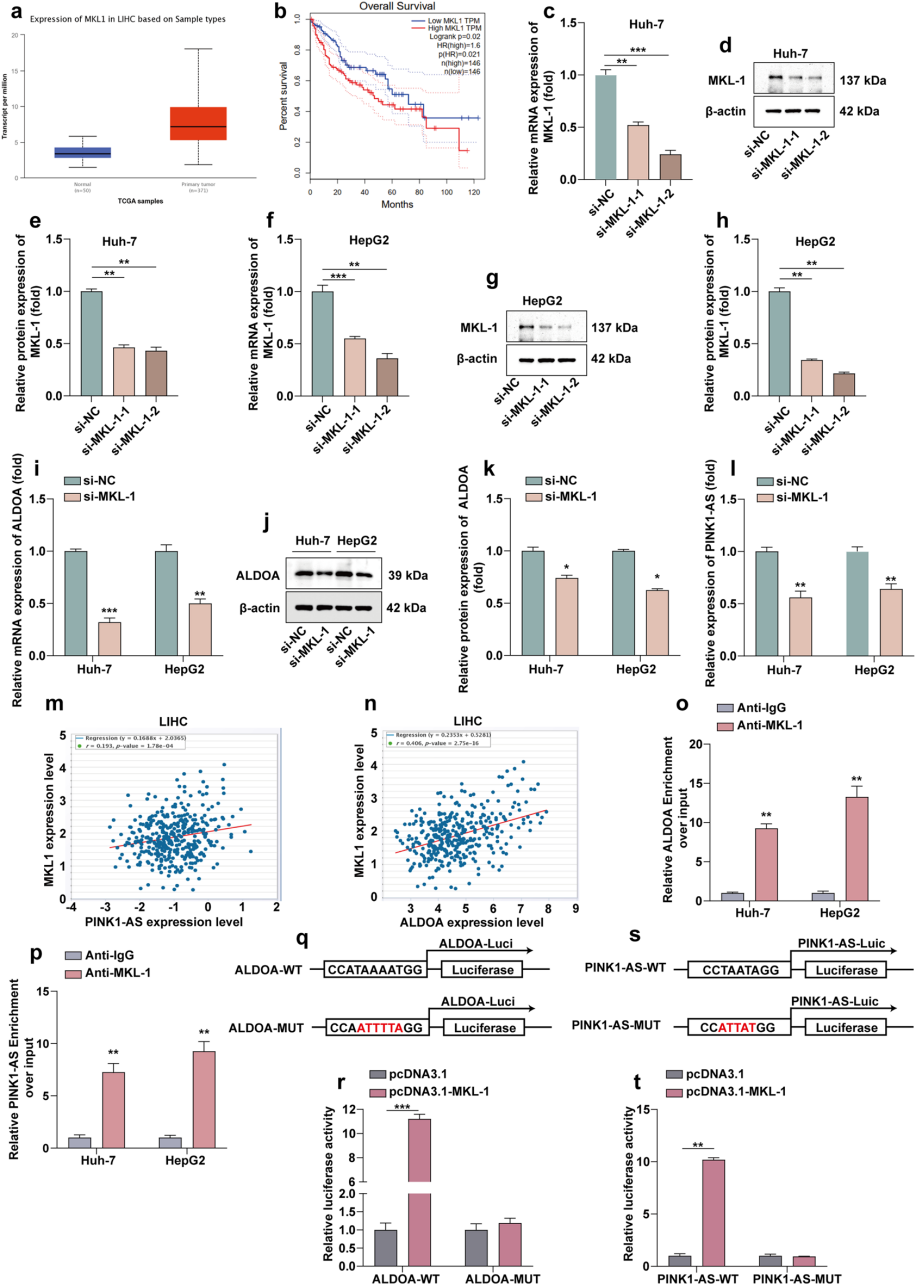
MKL-1 induces the expression of PINK1-AS and ALDOA as a transcription factor. (**a**) The box diagram shows the level of MKL-1 mRNA in the Ualcan database (http://ualcan.path.uab.edu/analysis.htm). (**b**) Kaplan–Meier survival curves showing the effect of MKL-1 on overall survival. (**c**–**h**) WB and RT-qPCR to verify the efficiency of siRNA knockdown of MKL-1. Statistical data are shown. (**i**,**g**,**k**) WB and RT-qPCR to determine the expression level of ALDOA in Huh-7 and HepG2 cells after knockdown of MKL-1. Statistical data are shown. (**l**) RT-qPCR determination of PINK1-AS expression levels in Huh-7 and HepG2 cells after knockdown of MKL-1. (**m**,**n**) Correlation analysis showed that MKL-1 was positively correlated with the expression of ALDOA and PINK1-AS (*P* < 0.001). (**o**,**p**) Quantitative ChIP analysis to show direct binding of MKL-1 to the endogenous PINK1-AS and ALDOA promoter regions. (**q**,**r**) Prediction of MKL-1 binding sites in the human PINK1-AS and ALDOA promoters by gene sequence analysis. (**s**,**t**) Luciferase reporter gene test to detect whether MKL-1 can target the PINK1-AS and ALDOA promoters. *P < 0.05; **P < 0.01; ***P < 0.001.

The promoter regions of PINK1-AS and ALDOA were located and analysed through the UCSC (https://genome.ucsc.edu/) website, the first 2000 bp upstream of the transcription start site was found to contain the MKL-1 binding site. Bioinformatics analysis shows that MKL-1 is up-regulated in HCC (Fig. 6a). The association between MKL-1 expression and survival outcomes of HCC patients was assessed using Kaplan–Meier survival curves, and analysis showed that patients with high MKL-1 expression had poor elevated overall survival (Fig. 6b). To verify whether MKL-1 mediated the expression of PINK1-AS and ALDOA, we knocked down MKL-1 using siRNA targeting MKL-1 (Fig. 6c–h). MKL-1 down-regulation resulted in a significant decrease in the expression of PINK1-AS and ALDOA in Huh-7 and HepG2 (Fig. 6i–l). Further, we found that silencing of MKL-1 significantly inhibited aerobic glycolysis in HCC cells (Fig. S5a,b). Correlation analysis showed that the expression level of MKL-1 was positively correlated with the expression levels of ALDOA and PINK1-AS in HCC tissues (Fig. 6m,n). To investigate whether MKL-1 directly transcriptionally regulates ALDOA and PINK1-AS expression, we have verified that MKL-1 can bind to the promoter regions of PINK1-AS and ALDOA by ChIP experiments (Fig. 6o,p). In addition, we constructed ALDOA and PINK1-AS promoter luciferase reporter plasmids (Fig. 6q,r). Luciferase assays showed that MKL-1 could promote PINK1-AS and ALDOA promoter luciferase activity (Fig. 6s,t).

## Discussion

The reprogramming of tumour metabolism secures favourable conditions for tumour cells to survive in a new environment^22,23^. The greater glycolytic capacity in highly malignant or metastatic tumour cells suggests a potential correlation between altered tumour metabolic conditions and their ability to proliferate and migrate invasively. Increasing ALDOA expression accelerates the process of glycolysis in tumour cells, while decreasing ALDOA expression decreases ATP production and reduces cell membrane integrity^24^. In addition to regulating glycolysis and energy metabolism, ALDOA can also be involved in tumourigenesis and development through a variety of pathways. In a hypoxic environment, ALDOA can mediate EMT in tumour cells by regulating hypoxia inducible factor-1α (HIF-1α), which enhances tumour cell migration^25,26^. Caspi M et al. concluded that ALDOA is a new regulator of the wnt signalling pathway that can GSK-3β-dependent mechanism to activate the classical wnt signaling pathway, which is closely related to tumorigenesis and development^27^. In this study, we used TCGA data to determine that ALDOA expression was upregulated in HCC. We then confirmed high ALDOA expression in HCC by RT-qPCR and WB analysis, high ALDOA expression in HCC tissues was associated with poor prognosis and may be an independent prognostic indicator. Through loss-of-function assays, we confirmed that ALDOA promotes glycolysis of HCC in vitro and in vivo. These results suggest that ALDOA may play an important role in the progression of HCC.

Studies have confirmed that dysregulation of miRNAs plays an important role in a variety of diseases, including malignancies and inflammatory diseases^28^. In previous studies, miR-34a-5p was found to have an important role in the pathogenesis of a variety of diseases. Gao J et al. found that miR-34a-5p was lowly expressed in colon cancer patients and could target P53 to promote tumour cell metastasis and disease recurrence^29^. Jiang et al. showed that garcinia cambogia could target miR-34a-5p through up-regulation of MDM4 gene to inhibit the anti-apoptotic ability of cancer cells in non-small cell lung cancer patients, thereby eliminating or shrinking cancer foci^30^. In addition, miR-34a-5p also has a regulatory role in the pathogenesis of many benign diseases. Cosín-Tomás et al. found that miR-34a-5p was differentially expressed in the serum of patients with Alzheimer's disease and normal subjects^31^. Li et al. showed that miR-34a-5p also has an important role in diabetic nephropathy, and high expression of miR-34a-5p inhibited cell proliferation and promote renal fibrosis^32^. In this study, after bioinformatics analysis, we found that ALDOA has a binding region to miR-34a-5p in its 3′UTR, and we hypothesized that ALDOA is likely to be a target gene of miR-34a-5p. Both protein expression assays and luciferase reporter experiment verified that ALDOA is a target gene of miR-34a-5p.

Current studies have shown that lncRNAs play an important role in the regulation of gene expression, embryonic development, species evolution, material metabolism, and tumourigenesis and metastasis^16,33–35^. PINK1-AS, an antisense RNA of PINK1, is regulated by PINK1-AS in the regulation of mitochondrial function. It has been reported that PINK1-AS expression is elevated in patients with multiple sclerosis^36^. Márki S et al. found that polymorphisms in the PINK1-AS gene were associated with Parkinson's disease^37^. In the present study, we found that PINK1-AS could act as a ceRNA to antagonize miR-34a-5p-mediated degradation of ALDOA and indirectly increase the expression of ALDOA mRNA. In vitro experiments also further demonstrated that PINK1-AS could promote aerobic glycolysis in HCC cells. However, under what circumstances will a lncRNAs digest or reserve the target miRNAs remain needs in-depth exploration.

MKL-1 is widely expressed in mammals and plays an important role in various physiological and pathological processes in the body^38^. Interfering with MKL-1 expression in breast cancer cells has been reported to attenuate the migration level of tumour cells^39^. The knockdown of MKL-1 in mice significantly reduced the tumorigenic ability of tumor cells and their ability to migrate to the lung with the blood circulation^40^. MKL-1 can also act as a transcription factor to promote cancer development by altering the expression of other genes in cancer cells. MKL-1 can bind directly to the core region of the CAPP1 promoter to regulate the activity of the CAPP1 promoter^41^. MKL-1 mediates TGF-β-induced RhoJ transcription to promote breast cancer cell migration and invasion^42^. In this study, we found that PINK1-AS and ALDOA are direct targets of the transcription factor MKL-1, as confirmed by the binding of MKL-1 to predicted sites in the PINK1-AS and ALDOA promoter regions and the significant induction of PINK1-AS and ALDOA promoter activity induced by MKL-1. Thus, the upregulation of PINK1-AS and ALDOA in HCC is partly attributed to MKL-1 activation during tumour progression.

In this study, we investigated the role and mechanism of ALDOA in HCC. We obtained that ALDOA is a target gene of miR-34a-5p through bioinformatic target analysis, and demonstrated that miR-34a-5p silences the expression of ALDOA by targeting its 3′UTR region, thus affecting glycolysis in HCC cells. PINK1-AS1 could act as a "sponge" for miR-34a-5p and thus inhibit the silencing effect of miR-34a-5p on ALDOA. We also found MKL-1 binding sites at the promoters of ALDOA and PINK1-AS, suggesting that MKL-1 promotes the transcription of ALDOA and PINK1-AS by targeting their promoters. In HCC cells, PINK1-AS and ALDOA levels are consistently parallel, with increased levels promoting aerobic glycolysis.

## Conclusion

In conclusion, we comprehensively investigated the functional role and molecular mechanism of ALDOA in HCC. Our results suggest that ALDOA is up-regulated in HCC cell lines and tissues. High ALDOA expression levels are an independent prognostic factor for the overall survival of HCC patients. Our study shows that MKL-1/PINK1-AS/miR-34a-5p/ALDOA form a circuit in cells to co-regulate glycolysis in HCC cells. Our findings support the idea that ALDOA plays a key role in HCC progression and is a potentially effective target for the treatment of HCC.

## Supplementary Information

Supplementary Information.

## Data Availability

The datasets used and/or analyzed during the present study are available from the corresponding author on reasonable request.

## References

[1] Bray F, Ferlay J, Soerjomataram I, Siegel R, Torre L, Jemal A (2018). Global cancer statistics 2018: GLOBOCAN estimates of incidence and mortality worldwide for 36 cancers in 185 countries. CA Cancer J. Clin..

[2] Liu Y, Zhang J, Qin Y, Wang W, Wei L, Teng Y (2013). PROX1 promotes hepatocellular carcinoma metastasis by way of up-regulating hypoxia-inducible factor 1α expression and protein stability. Hepatology.

[3] Hartke J, Johnson M, Ghabril M (2017). The diagnosis and treatment of hepatocellular carcinoma. Semin. Diagn. Pathol..

[4] Sim H, Knox J (2018). Hepatocellular carcinoma in the era of immunotherapy. Curr. Probl. Cancer.

[5] Gatenby R, Gillies R (2004). Why do cancers have high aerobic glycolysis?. Nat. Rev. Cancer.

[6] Choudhary K, Rohatgi N, Halldorsson S, Briem E, Gudjonsson T, Gudmundsson S (2016). EGFR signal-network reconstruction demonstrates metabolic crosstalk in EMT. PLoS Comput. Biol..

[7] Xia S, Lin R, Jin L, Zhao L, Kang H, Pan Y (2017). Prevention of dietary-fat-fueled ketogenesis attenuates BRAF V600E tumor growth. Cell Metab..

[8] Li W, Pan T, Jiang W, Zhao H (2020). HCG18/miR-34a-5p/HMMR axis accelerates the progression of lung adenocarcinoma. Biomed. Pharmacother..

[9] Li S, Zhu K, Liu L, Gu J (2020). lncARSR sponges miR-34a-5p to promote colorectal cancer invasion and metastasis via hexokinase-1-mediated glycolysis. Cancer Sci..

[10] Bartel D (2009). MicroRNAs: Target recognition and regulatory functions. Cell.

[11] Lin S, Gregory RI (2015). MicroRNA biogenesis pathways in cancer. Nat. Rev. Cancer.

[12] Song JH, Meltzer SJ (2012). MicroRNAs in pathogenesis, diagnosis, and treatment of gastroesophageal cancers. Gastroenterology.

[13] Abraham JM, Meltzer SJ (2017). Long noncoding RNAs in the pathogenesis of Barrett's esophagus and esophageal carcinoma. Gastroenterology.

[14] Guttman M, Rinn JL (2012). Modular regulatory principles of large non-coding RNAs. Nature.

[15] Palmieri F, Monné M (2016). Discoveries, metabolic roles and diseases of mitochondrial carriers: A review. Biochim. Biophys. Acta.

[16] Beermann J, Piccoli MT, Viereck J, Thum T (2016). Non-coding RNAs in development and disease: Background, mechanisms, and therapeutic approaches. Physiol. Rev..

[17] Harries LW (2012). Long non-coding RNAs and human disease. Biochem. Soc. Trans..

[18] Scheele C, Petrovic N, Faghihi MA, Lassmann T, Fredriksson K, Rooyackers O (2007). The human PINK1 locus is regulated in vivo by a non-coding natural antisense RNA during modulation of mitochondrial function. BMC Genomics.

[19] Wang K, Li J, Xiong YF, Zeng Z, Zhang X, Li HY (2018). A potential prognostic long noncoding RNA signature to predict recurrence among ER-positive breast cancer patients treated with tamoxifen. Sci. Rep..

[20] Lambert SA, Jolma A, Campitelli LF, Das PK, Yin Y, Albu M, Chen X, Taipale J, Hughes TR, Weirauch MT (2018). The human transcription factors. Cell.

[21] Olson EN, Nordheim A (2010). Linking actin dynamics and gene transcription to drive cellular motile functions. Nat. Rev. Mol. Cell Biol..

[22] Deberardinis RJ, Chandel NS (2016). Fundamentals of cancer metabolism. Sci. Adv..

[23] Gentric G, Mieulet V, Mechtagrigoriou F (2017). Heterogeneity in cancer metabolism: New concepts in an old field. Antioxid. Redox Signal..

[24] Grandjean G, de Jong PR (2016). Definition of a novel feed-forward mechanism for glycolysis-HIF1α signaling in hypoxic tumors highlights aldolase a as a therapeutic target. Cancer Res..

[25] Ji S, Zhang B, Liu J, Qin Y, Liang C, Shi S (2016). ALDOA functions as an oncogene in the highly metastatic pancreatic cancer. Cancer Lett..

[26] Jiang Z, Wang X, Jing L, Yang H, Lin X (2018). Aldolase A as a prognostic factor and mediator of progression via inducing epithelial-mesenchymal transition in gastric cancer. J. Cell. Mol. Med. Rep..

[27] Caspi M, Perry G, Skalka N, Meisel S, Firsow A, Amit M, Rosin-Arbesfeld R (2014). Aldolase positively regulates of the canonical Wnt signaling pathway. Mol. Cancer.

[28] Vishnoi A, Rani S (2017). MiRNA biogenesis and regulation of diseases: An overview. Methods Mol. Biol..

[29] Gao J, Li N, Dong Y, Li S, Xu L, Li X (2015). miR-34a-5p suppresses colorectal cancer metastasis and predicts recurrence in patients with stage II/III colorectal cancer. Oncogene.

[30] Jiang ZQ, Li MH, Qin YM, Jiang HY, Zhang X, Wu MH (2018). Luteolin inhibits tumorigenesis and induces apoptosis of non-small cell lung cancer cells via regulation of microRNA-34a-5p. Int. J. Mol. Sci..

[31] Cosín-Tomás M, Antonell A, Lladó A, Alcolea D, Fortea J, Ezquerra M (2017). Plasma miR-34a-5p and miR-545-3p as early biomarkers of Alzheimer's disease: Potential and limitations. Mol. Neurobiol..

[32] Li A, Peng R, Sun Y, Liu H, Peng H, Zhang Z (2018). LincRNA 1700020I14Rik alleviates cell proliferation and fibrosis in diabetic nephropathy via miR-34a-5p/Sirt1/HIF-1α signaling. Cell Death Dis..

[33] Calle AS, Kawamura Y, Yamamoto Y, Takeshita F, Ochiya T (2018). Emerging roles of long non-codingRNAin cancer. Cancer Sci..

[34] Jarroux J, Morillon A, Pinskaya M (2017). History, discovery, and classification of lncRNAs. Adv. Exp. Med. Biol..

[35] Dykes IM, Emanueli C (2017). Transcriptional and post-transcriptional gene regulation by long non-coding RNA. Genomics Proteomics Bioinform..

[36] Patoughi M, Ghafouri-Fard S, Arsang-Jang S, Taheri M (2020). Expression analysis of PINK1 and PINK1-AS in multiple sclerosis patients versus healthy subjects. Nucleosides Nucleotides Nucl. Acids Res..

[37] Márki S, Gbls A, Szlávicz E, Trk N, Széll M (2018). The rs13388259 intergenic polymorphism in the genomic context of the BCYRN1 gene is associated with Parkinson's disease in the Hungarian population. Parkinsons Dis..

[38] Cheng EC (2009). Role for MKL1 in Megakaryocytic Maturation.

[39] Gurbuz I, Ferralli J, Roloff T, Chiquet-Ehrismann R, Asparuhova MB (2014). SAP domain-dependent Mkl1 signaling stimulates proliferation and cell migration by induction of a distinct gene set indicative of poor prognosis in breast cancer patients. Mol. Cancer.

[40] Cheng X, Yang Y, Fan Z, Yu L, Bai H, Zhou B (2015). MKL1 potentiates lung cancer cell migration and invasion by epigenetically activating MMP9 transcription. Oncogene.

[41] Zhang HM, Li H, Wang GX, Wang J, Liao XH (2020). MKL1/miR-5100/CAAP1 loop regulates autophagy and apoptosis in gastric cancer cells. Neoplasia.

[42] Chen B, Yuan Y, Sun L, Chen J, Xu Y (2020). MKL1 mediates TGF-β induced RhoJ transcription to promote breast cancer cell migration and invasion. Front. Cell Dev. Biol..

