# Supplementary material for: MKL-1-induced PINK1-AS overexpression contributes to the malignant progression of hepatocellular carcinoma via ALDOA-mediated glycolysis
Source: Sci Rep. 2022 Dec 9;12:21283. doi: 10.1038/s41598-022-24023-w (PMC9734095; doi:10.1038/s41598-022-24023-w)

**Supplementary figure legends**

**
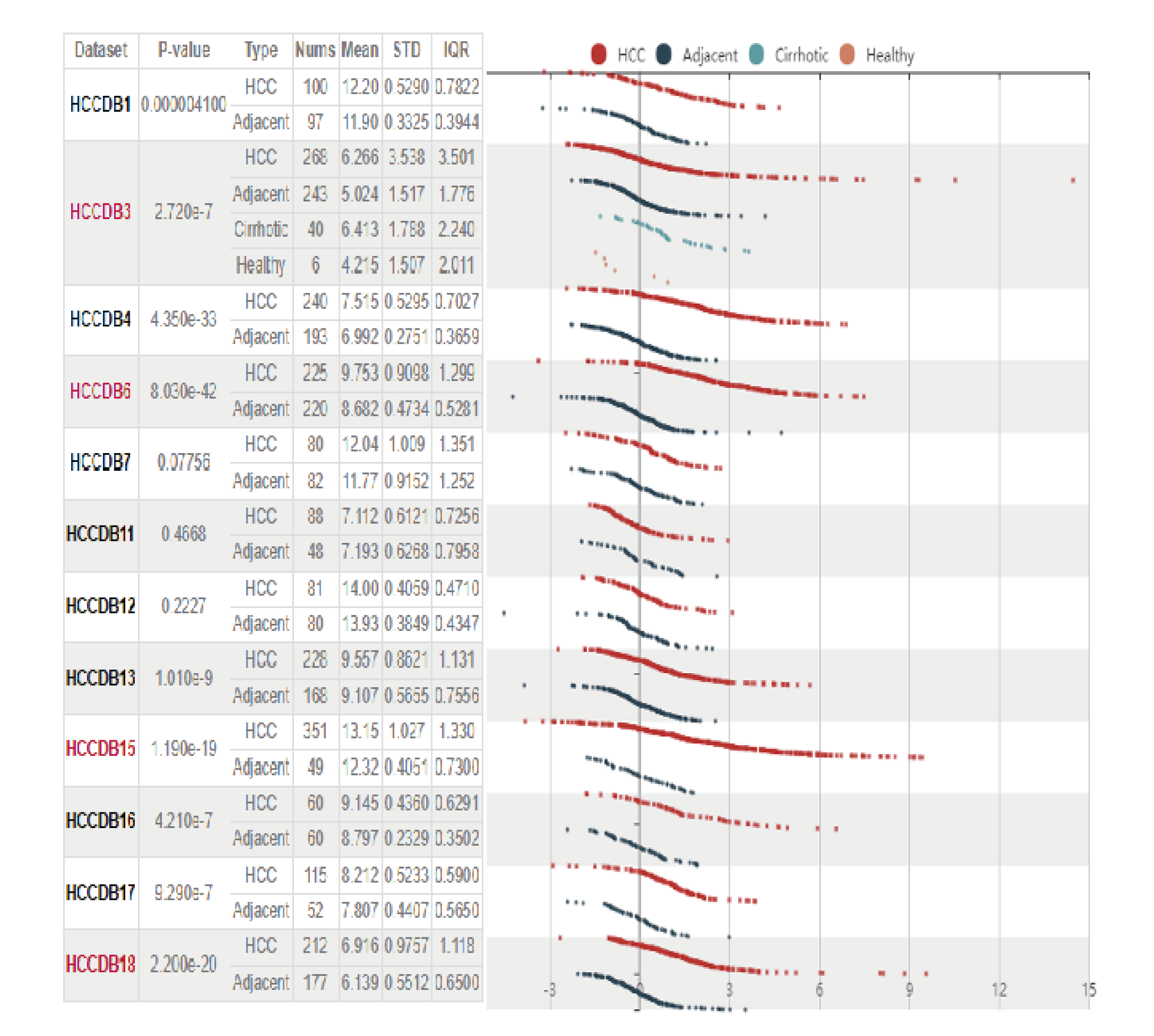
**

Fig. S1. According to t test in HCCDB, the expression of ALDOA in tumor tissue and adjacent normal tissue was revealed.


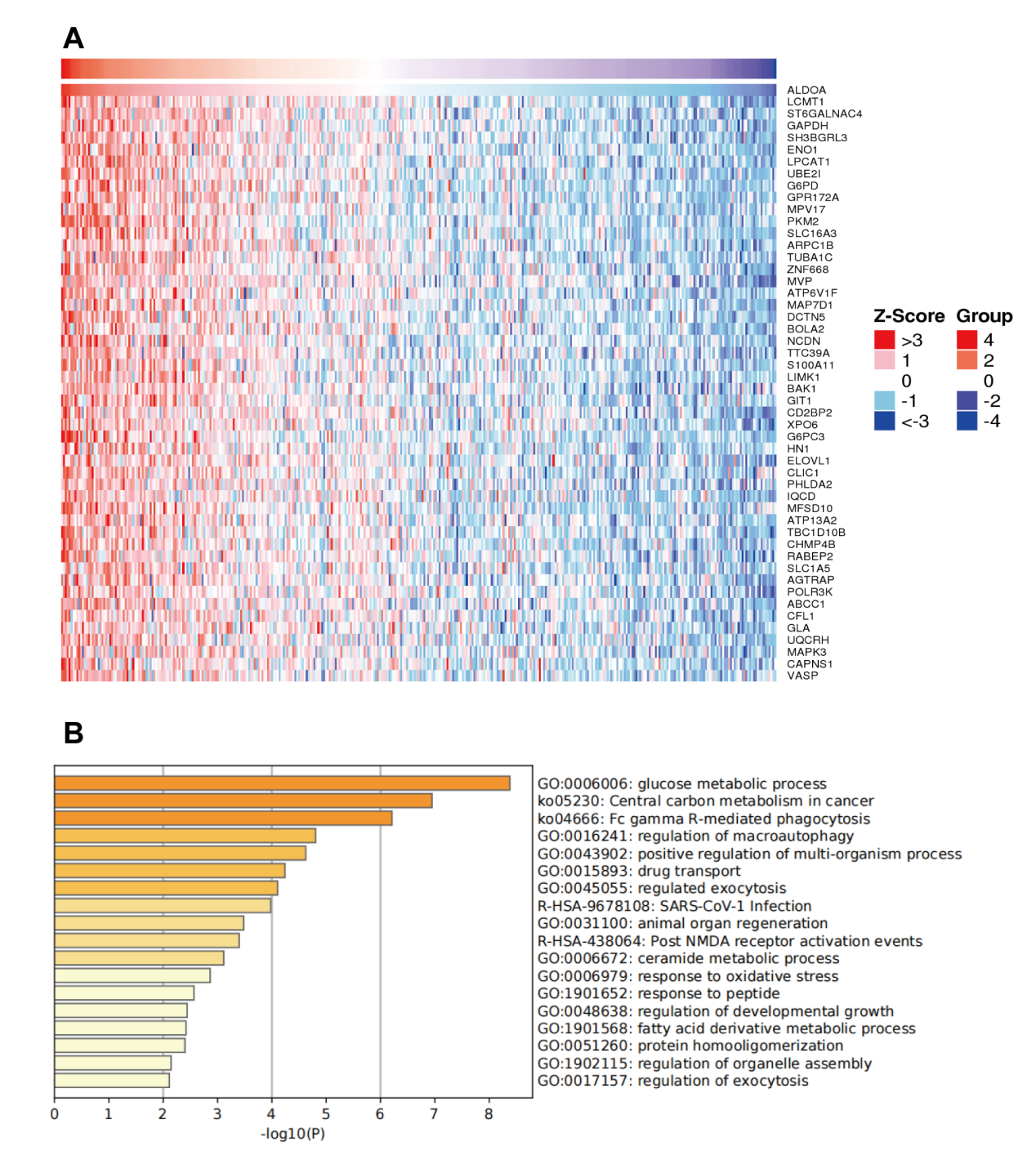


**Fig. S2. ALDOA co-expression genes in HCC.** (a) Heat maps showing top 50 genes positively correlated with ALDOA in LIHC. (b) Functional enrichment analysis of ALDOA in the LIHC cohort.


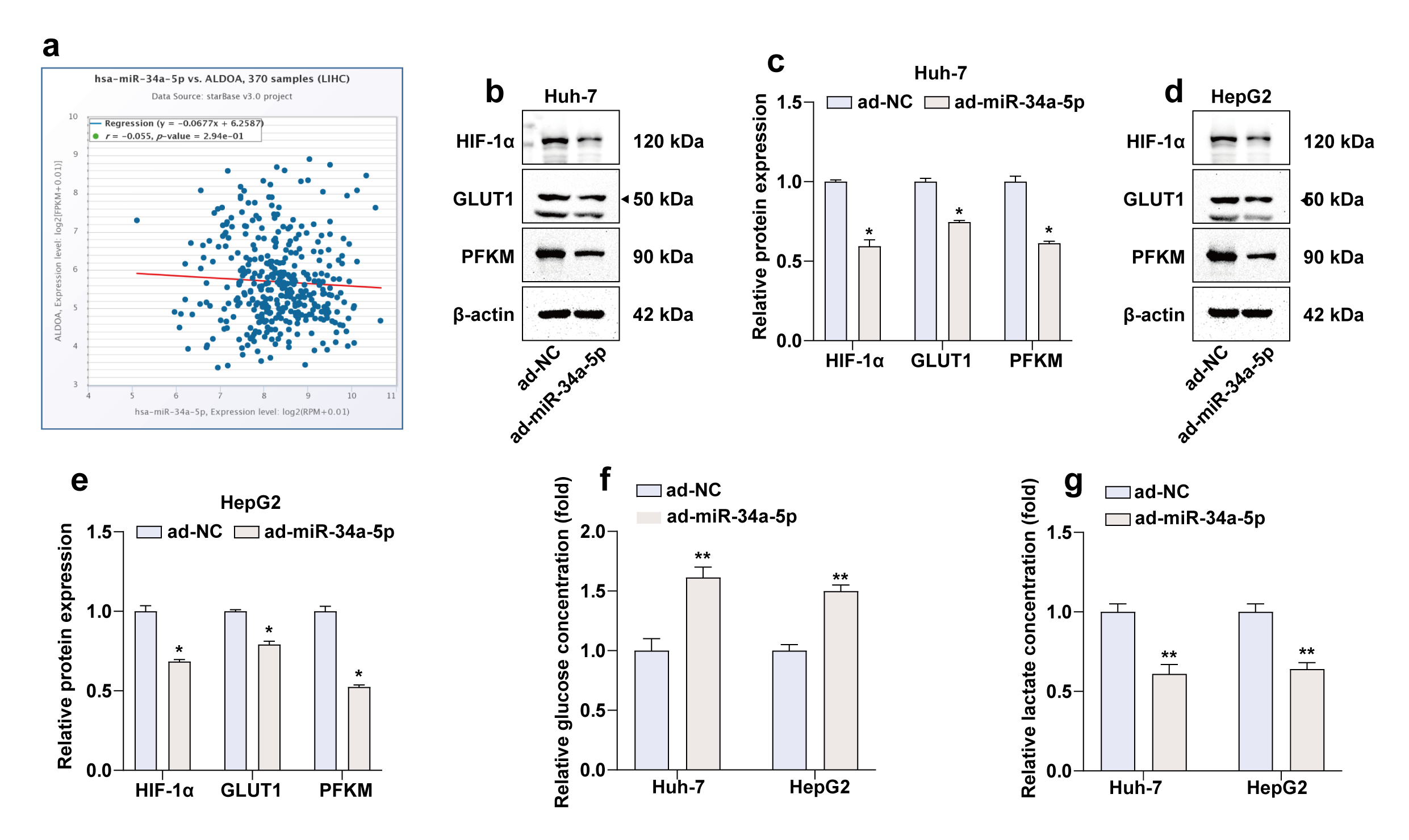


**Fig. S3. MiR-34a-5p targets ALDOA inhibits aerobic glycolysis of HCC cells.** (a) Correlation analysis showing a negative correlation between miR-34a-5p and ALDOA expression. (b, c, d, e) WB detection of aerobic glycolysis-related protein expression levels after overexpression of miR-34a-5p. (f, g) Glucose and lactate levels in the culture medium supernatants of two HCC cell lines after overexpression of miR-34a-5p were detected by the kit.


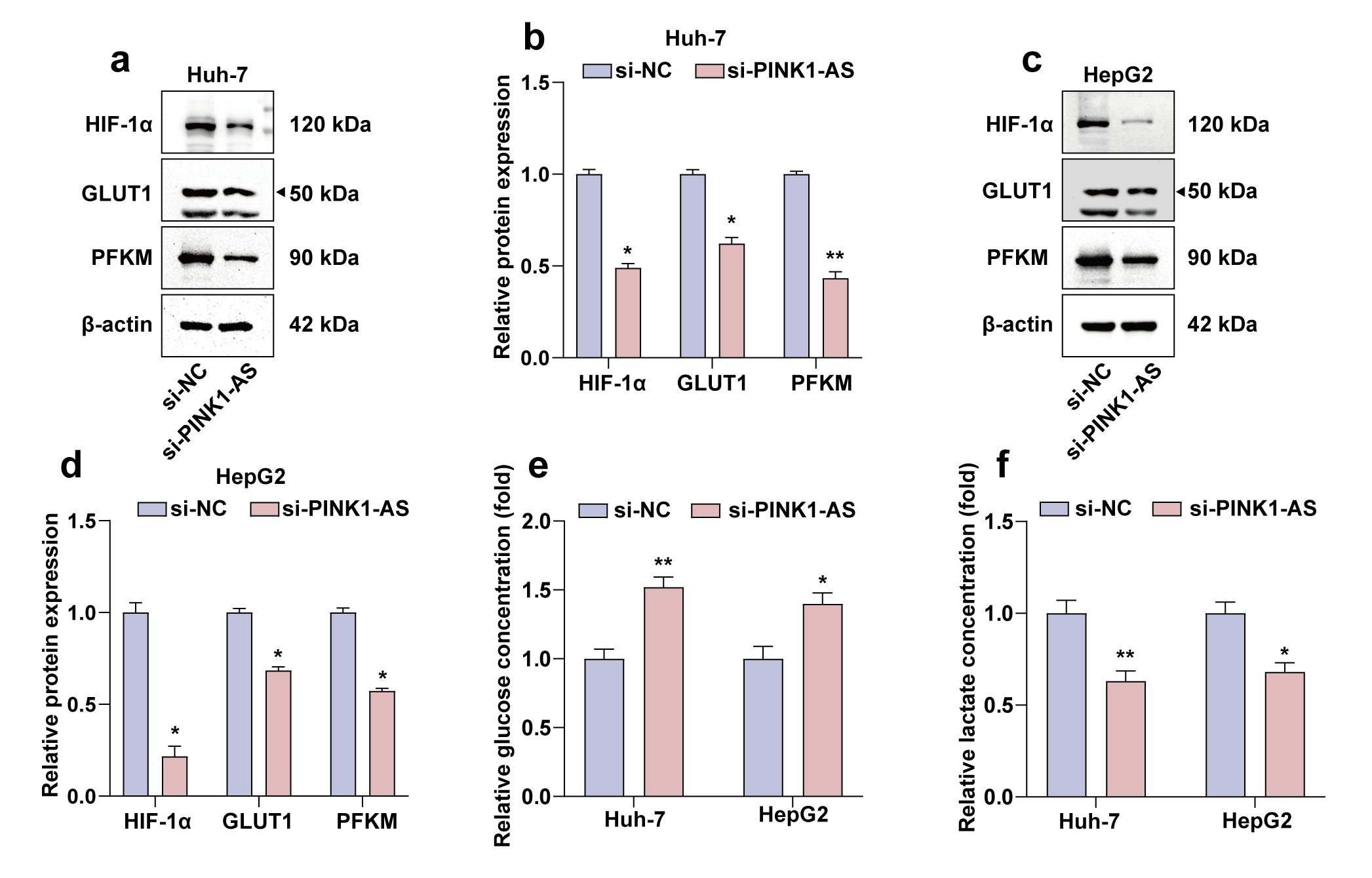


**Fig. S4.** **PINK1-AS targets miR-34a-5p promote aerobic glycolysis of HCC cells.** (a, b, c, d) WB detection of aerobic glycolysis-related protein expression levels after knockdown of PINK1-AS. (e, f) Glucose and lactate levels in the culture medium supernatants of two HCC cells after knockdown of PINK1-AS were detected by the kit.


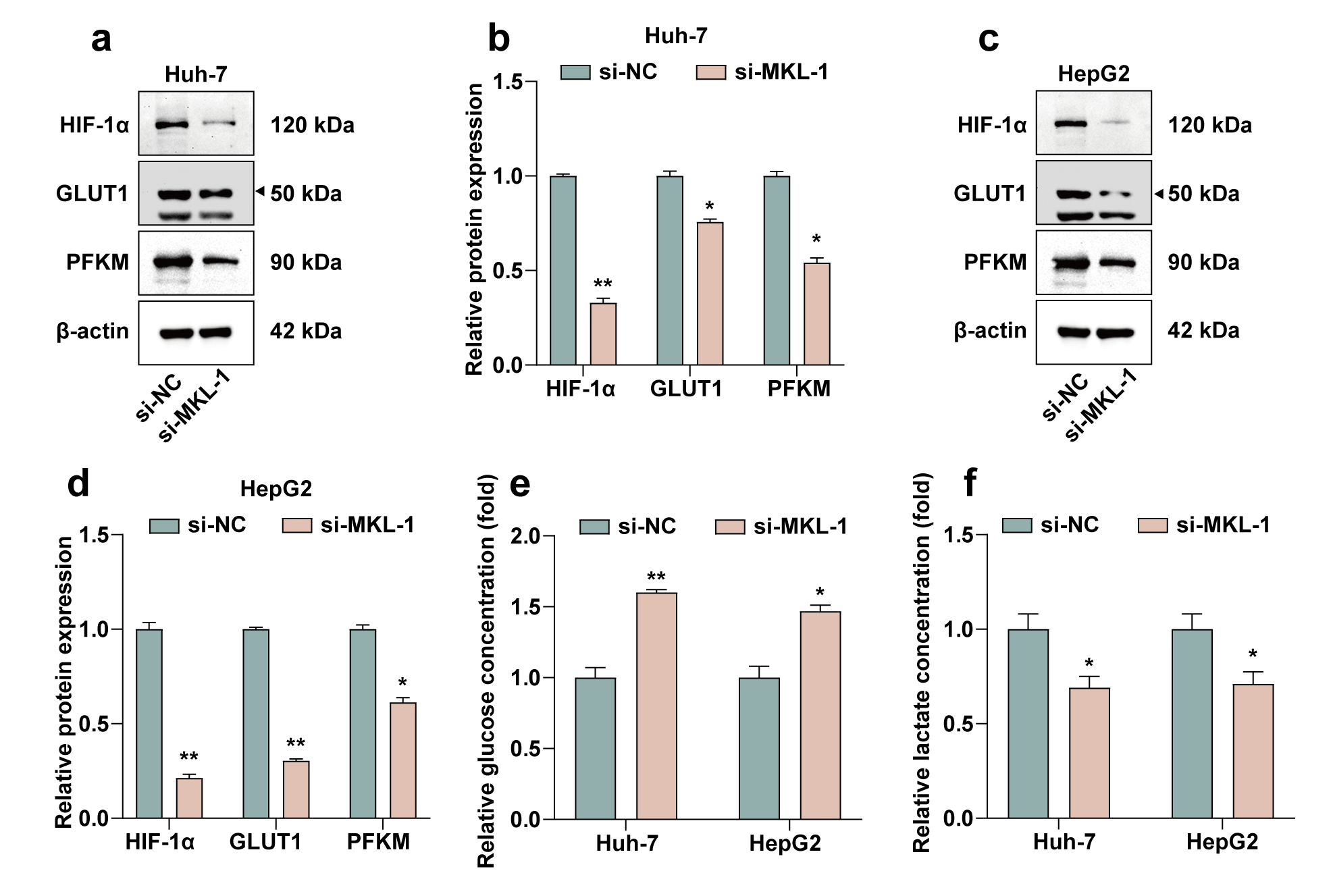


**Fig. S5. MKL-1 promotes the aerobic glycolysis process of HCC cells.** (a, b, c, d) WB detection of aerobic glycolysis-related protein expression levels after knockdown of MKL-1. (e, f) Glucose and lactate levels in the culture medium supernatants of two HCC cells after knockdown of PINK1-AS were detected by the kit.

**Supplementary Tables**

**Supplementary Table 1**

| β-actin | Forward | 5'-CATGTACGTTGCTATCCAGGC-3' |
| --- | --- | --- |
|  | Reverse | 5'-CTCCTTAATGTCACGCACGAT-3' |
| ALDOA | Forward | 5'-ATGCCCTACCAATATCCAGCA-3' |
|  | Reverse | 5'-GCTCCCAGTGGACTCATCTG-3' |
| PINK1-AS | Forward | 5'-CCGATTGTTGGAGGAGCCAG-3' |
|  | Reverse | 5'-AGCAGGCTTTGGGTTGAGAC-3' |
| MKL-1 | Forward | 5'-ATGCCGCCTTTGAAAAGTCCA-3' |
|  | Reverse | 5'-TCTTCCGTTTGAGATAGTCCTCT-3' |
| miR-34a-5p | Forward | 5'-ACACTCCAGCTGGGTGGCAGTGTCTTAGC-3’ |
|  | Reverse | 5'-CTCAACTGGTGTCGTGGAGTCGGCAATTCAGTTGAGACAACCA-3' |
| U6 | Forward | 5'-CTCGCTTCGGCAGCACA-3' |
|  | Reverse | 5'-AACGCTTTCACGAATTTGCGT-3' |

Table 1: Primers of RT-qPCR

**Supplementary Table 2**

| si-1 ALDOA | 5’-GACAAATGGCGAGACTACCACCAA-3’ |
| --- | --- |
| si-2 ALDOA | 5’-CCAGTATCTGCCAGCAGAATGGCAT-3’ |
| sh-ALDOA | 5'-CCGGGCATCCATCAACCTCAATGCTCTCGAGAGCATTGAGGTTGATGGATGCTTTTTG-3' |
| si-PINK1-AS | 5’-AATGAAGATGCTCTTTCTGGC-3’ |
| si-1 MKL-1 | 5’-ACTCTACTGGAACCTGAGATGTTAA-3’ |
| si-2 MKL-1 | 5’-GACTCTACTGGAACCTGAGATGTTA-3’ |
| miR-34a-5p inhibitor | 5’-ACAACCAGCUAAGACACUGCCA-3’ |
| miR-34a-5p mimics | 5’-UGGCAGUGUCUUAGCUGGUUGU-3’ |

Table 2: Sequence of siRNAs, shRNAs, miR-34a-5p inhibitor and miR-34a-5p mimics.

**Supplementary Table 3**

| ALDOA | Forward | 5'-TGATGGACAGAAAAATGTGTGTGCT-3' |
| --- | --- | --- |
|  | Reverse | 5'-GGGTACTGTCCATCATTCCCAGGAA-3' |
| PINK1-AS | Forward | 5'-AGCTGTGGCTCAGACCCAGAAGGGG-3' |
|  | Reverse | 5'-TTCCCCCACATGTCCACTGAATGCA-3' |

Table 3: Sequences of Chip

**Original Blots**


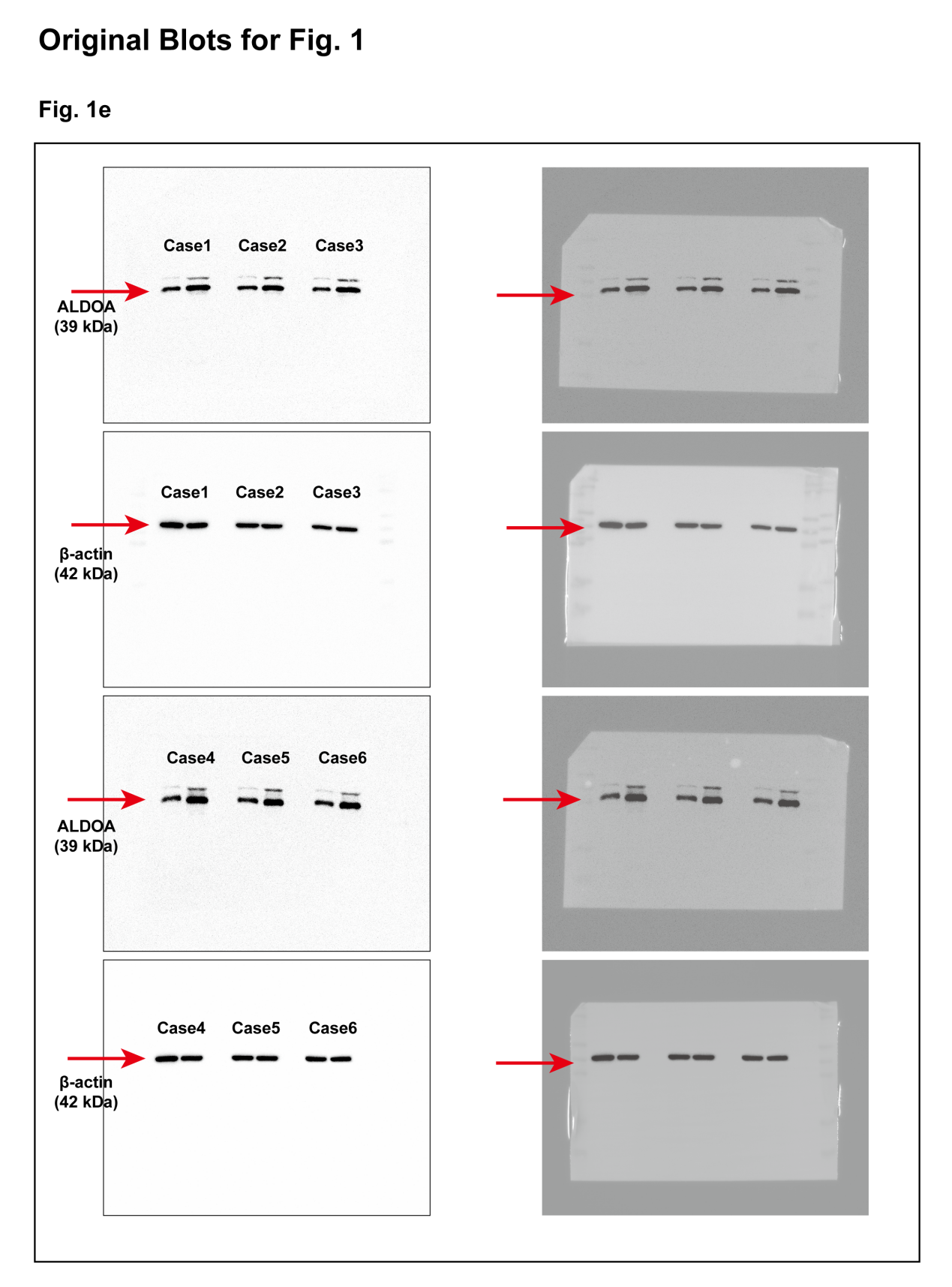


**Original Blots**


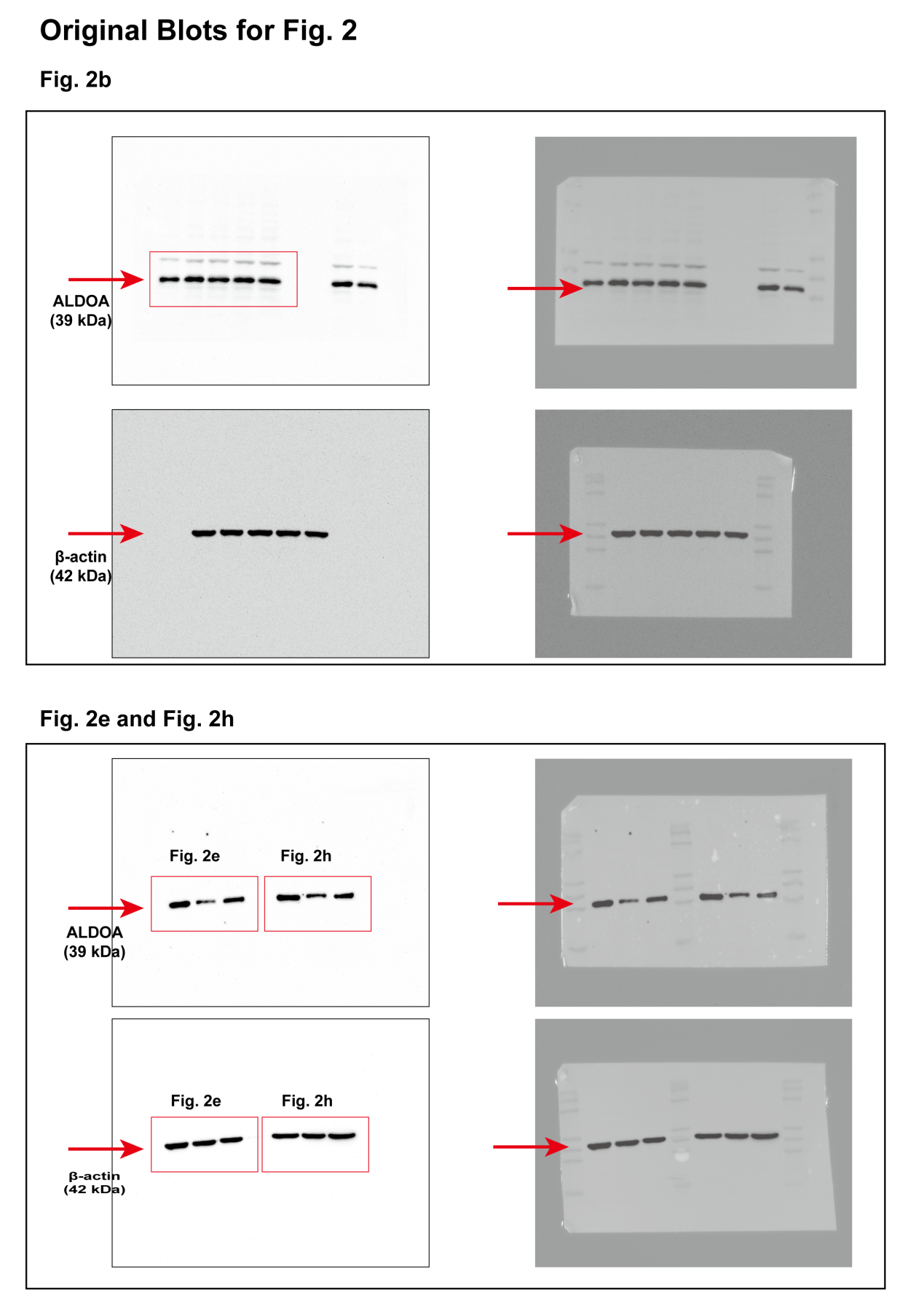


**Original Blots**


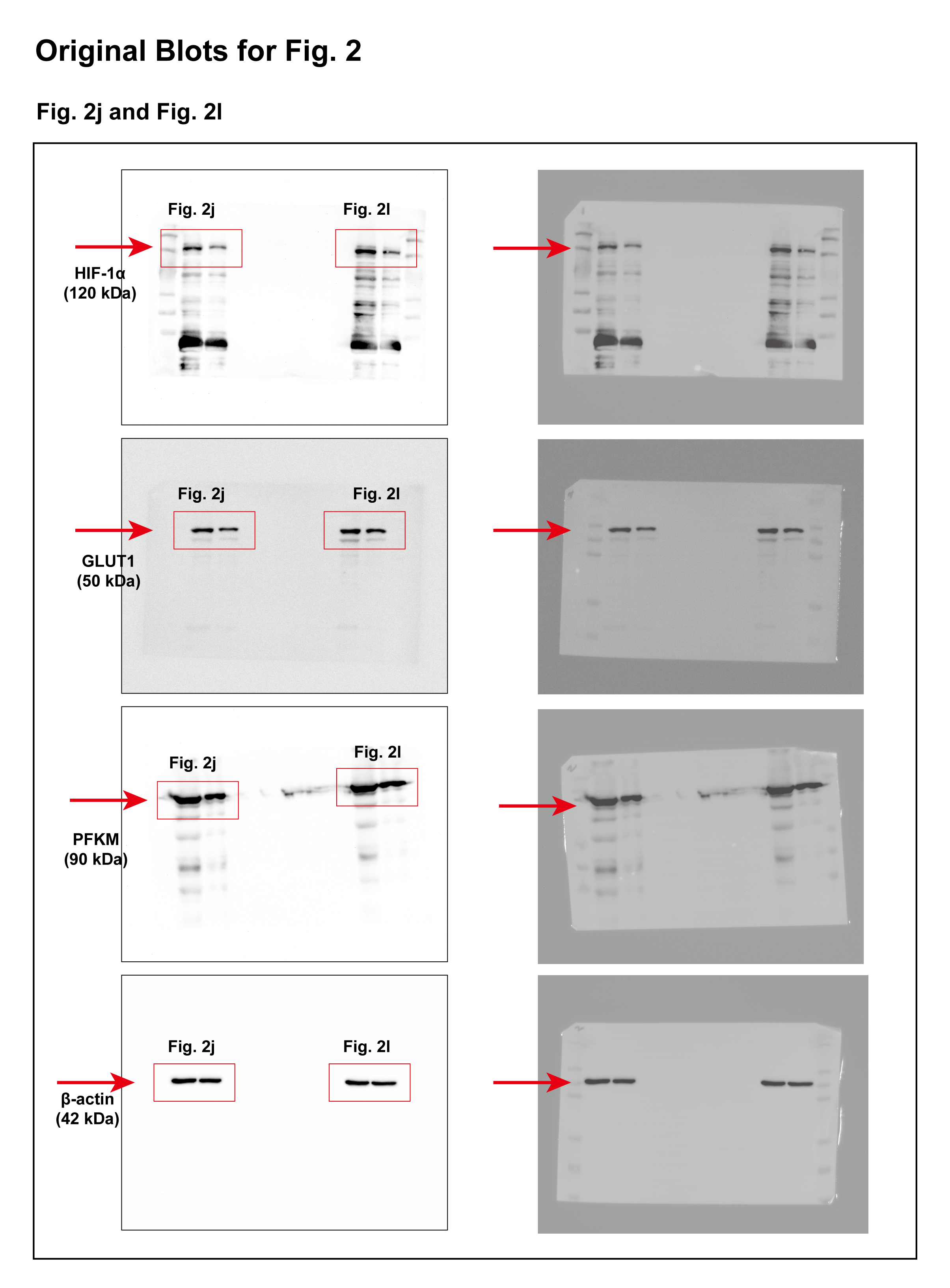


**Original Blots**


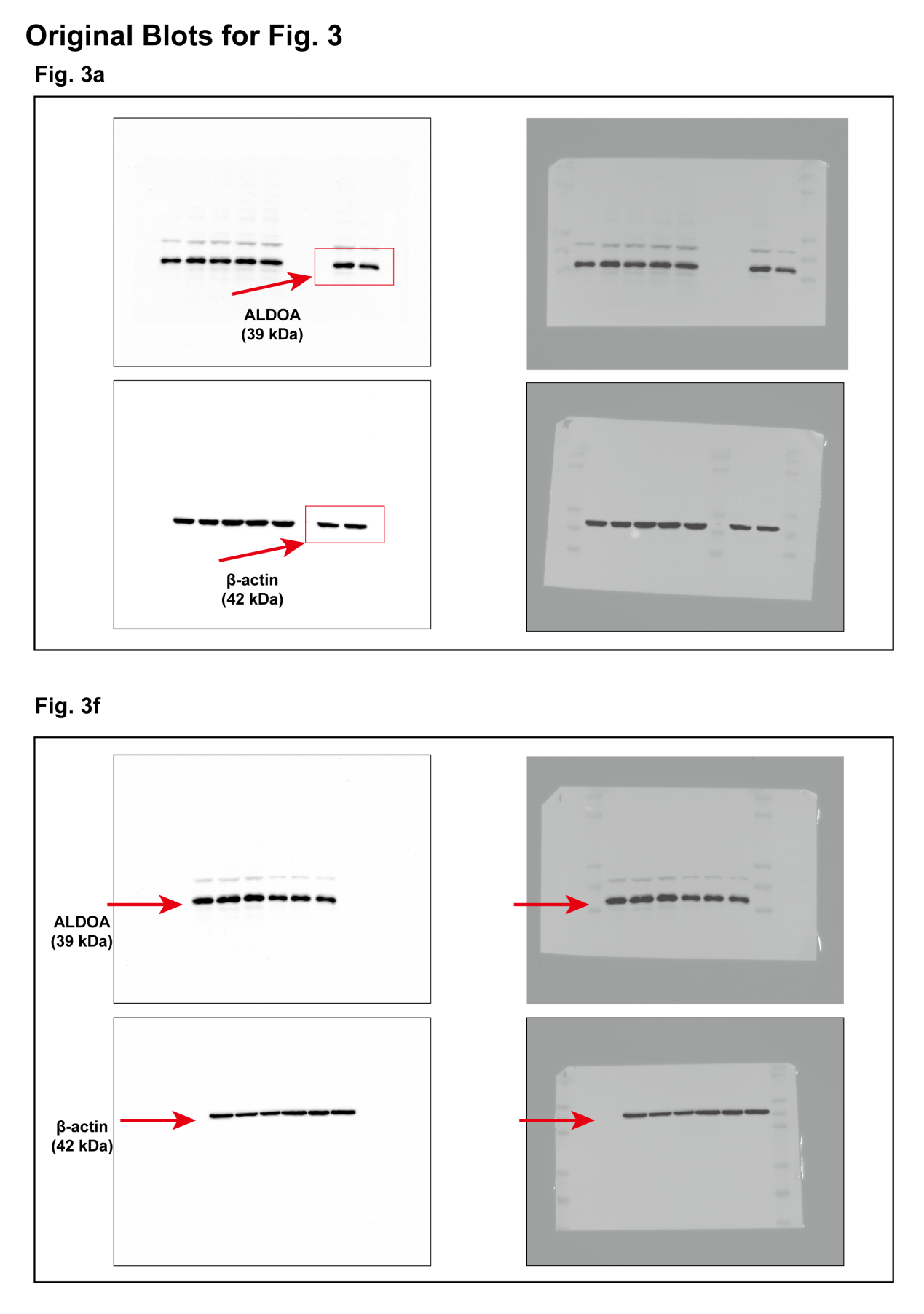


**Original Blots**


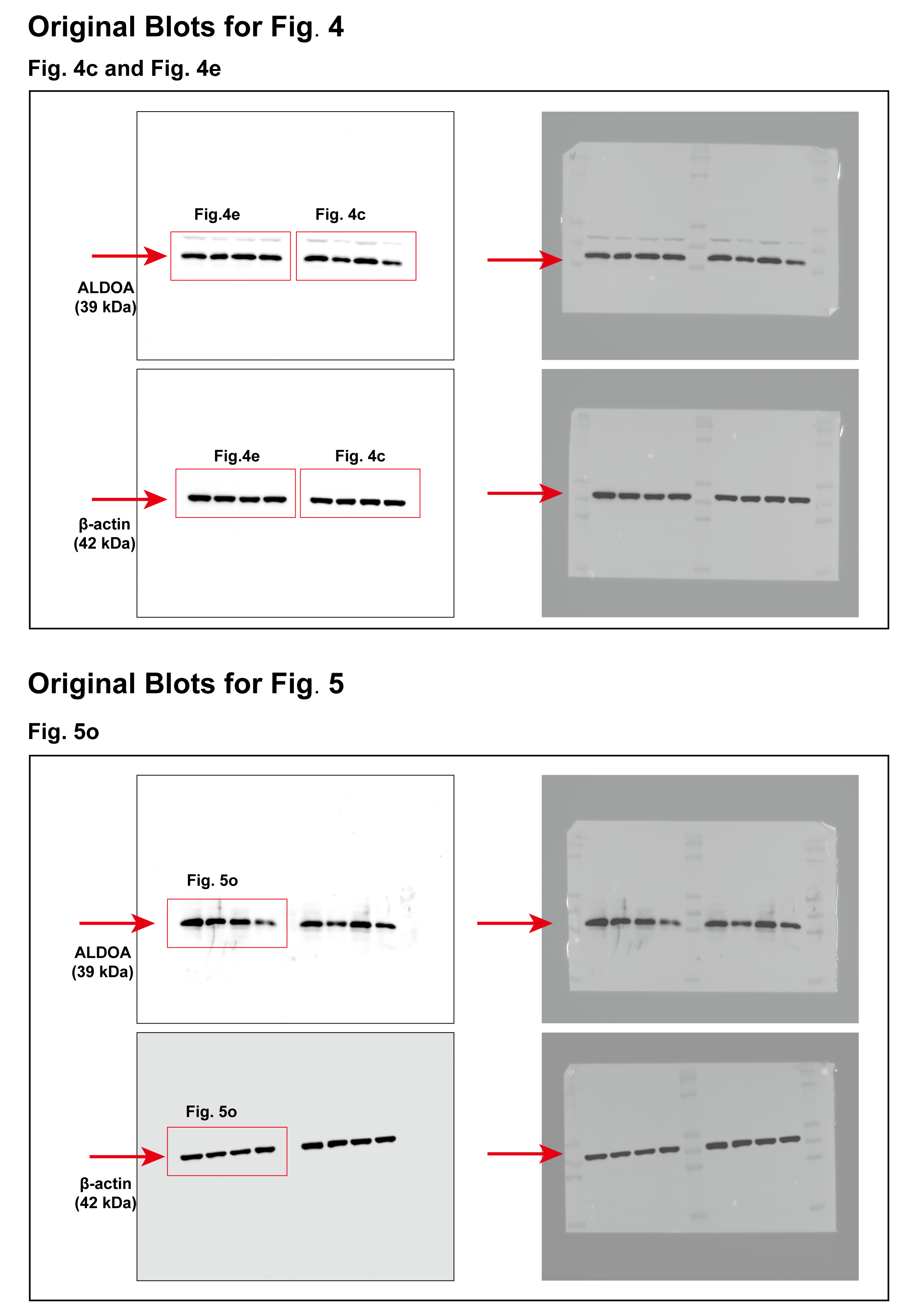


**Original Blots**


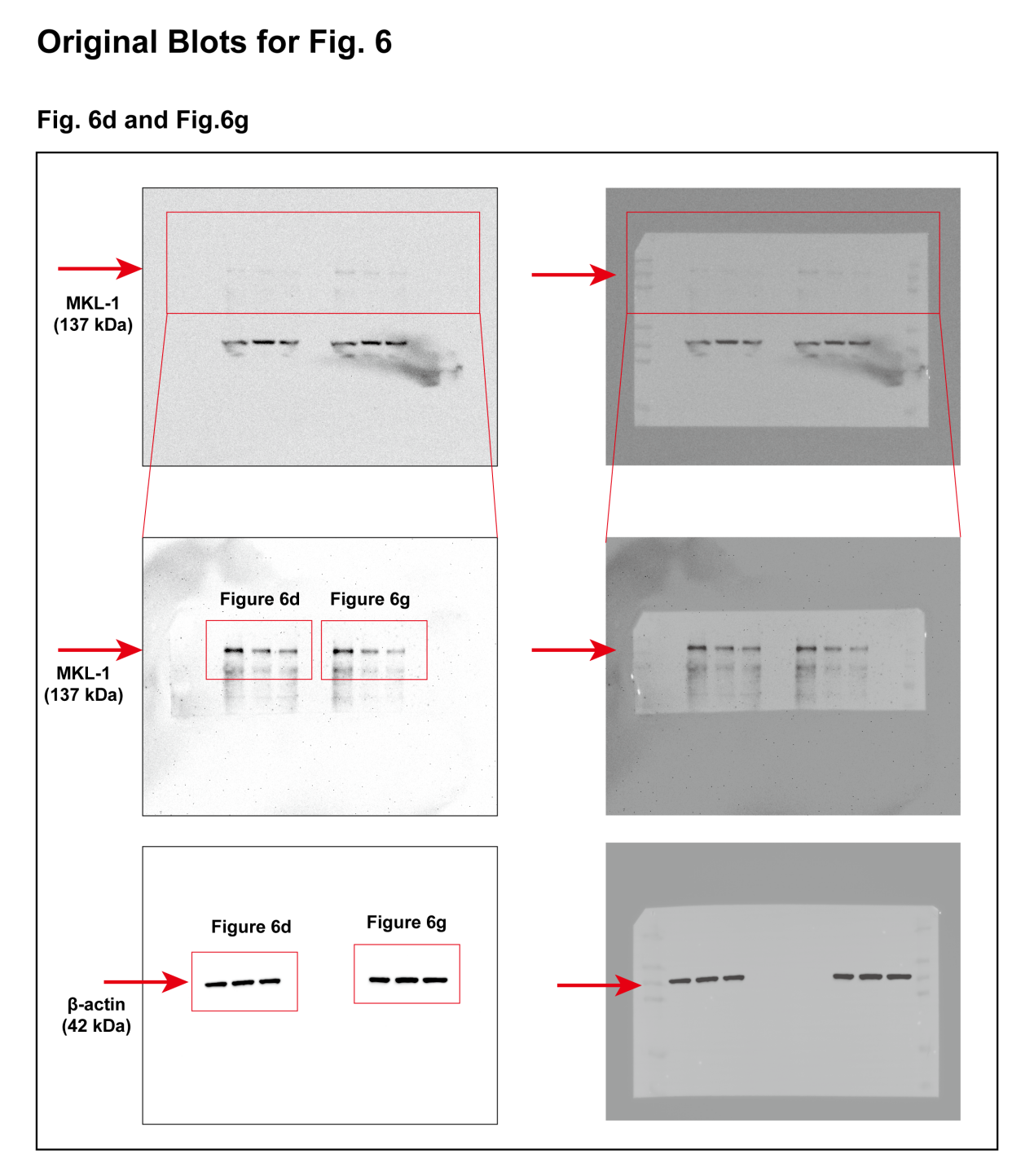


**Original Blots**


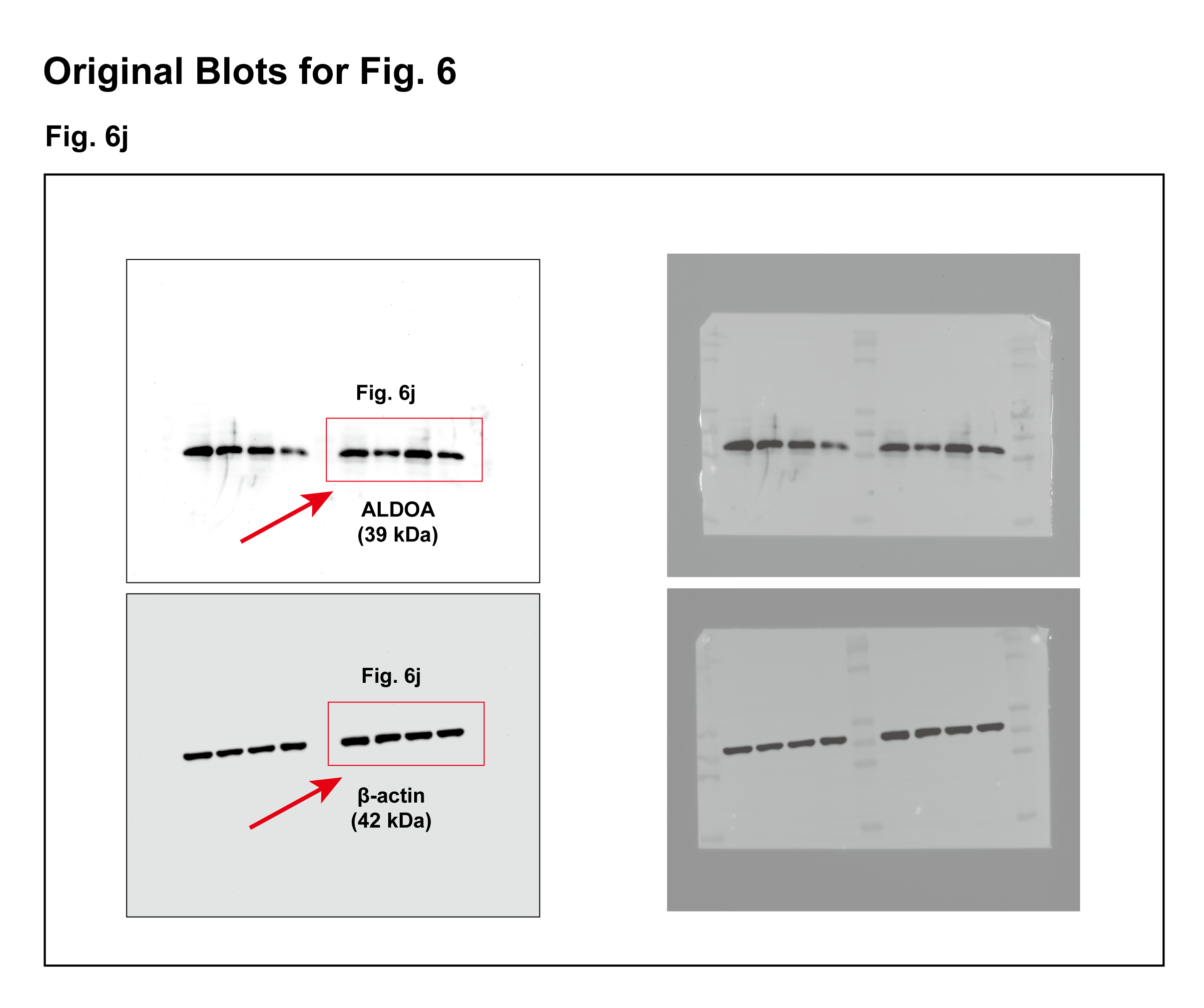


**Original Blots**


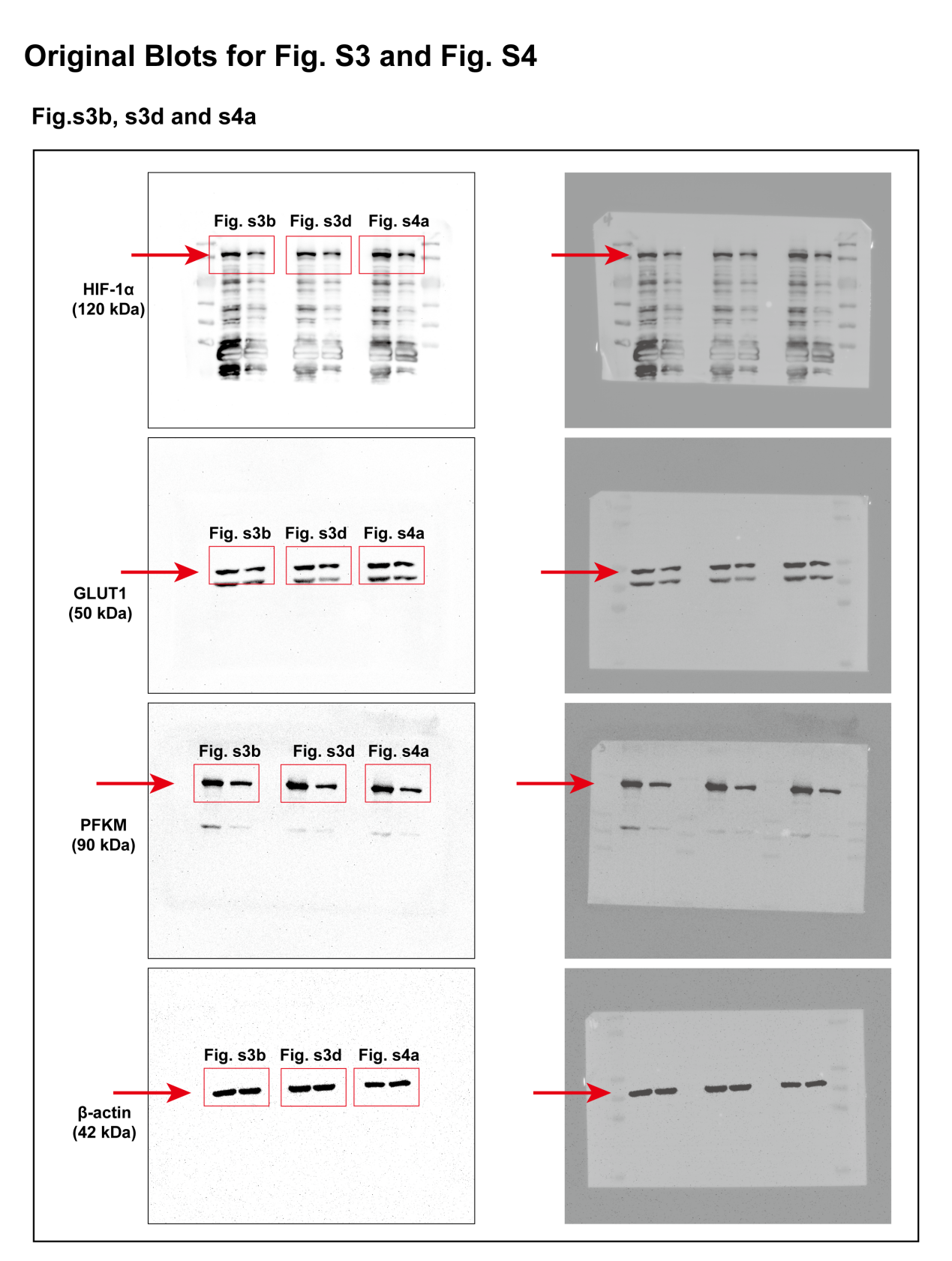


**Original Blots**


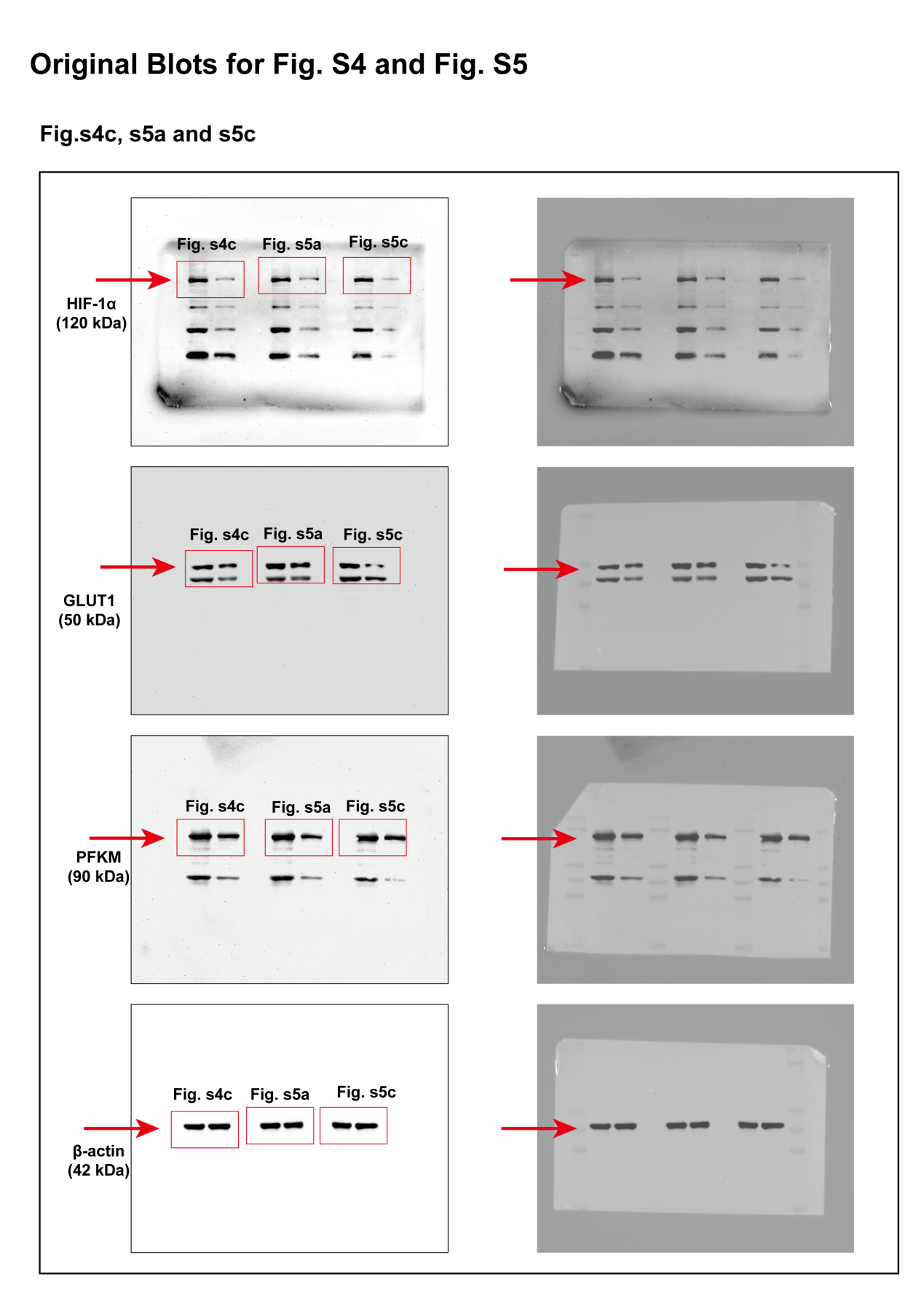

Supplement: Supplementary file 1 — Supplementary Information. [file 41598_2022_24023_MOESM1_ESM.docx]
